# Novel insight into the origin of the growth dynamics of sauropod dinosaurs

**DOI:** 10.1371/journal.pone.0179707

**Published:** 2017-06-27

**Authors:** Ignacio Alejandro Cerda, Anusuya Chinsamy, Diego Pol, Cecilia Apaldetti, Alejandro Otero, Jaime Eduardo Powell, Ricardo Nestor Martínez

**Affiliations:** 1 CONICET-Instituto de Investigaciónen Paleobiología y Geología, Universidad Nacional de Río Negro, Museo Carlos Ameghino, Cipolletti, Río Negro, Argentina; 2 University of Cape Town, Department of Biological Sciences, South Africa; 3 CONICET- Museo Paleontológico Egidio Feruglio, Trelew, Chubut, Argentina; 4 IMCN-Instituto y Museo de Ciencias Naturales, Universidad Nacional de San Juan, San Juan, Argentina; 5 CONICET-División Paleontología de Vertebrados, Museo de La Plata, La Plata, Argentina; 6 CONICET-Facultad de Ciencias Naturales Universidad Nacional de Tucumán, Tucumán, Argentina; College of the Holy Cross, UNITED STATES

## Abstract

Sauropod dinosaurs include the largest terrestrial animals and are considered to have uninterrupted rapid rates of growth, which differs from their more basal relatives, which have a slower cyclical growth. Here we examine the bone microstructure of several sauropodomorph dinosaurs, including basal taxa, as well as the more derived sauropods. Although our results agree that the plesiomorphic condition for Sauropodomorpha is cyclical growth dynamics, we found that the hypothesized dichotomy between the growth patterns of basal and more derived sauropodomorphs is not supported. Here, we show that sauropod-like growth dynamics of uninterrupted rapid growth also occurred in some basal sauropodomorphs, and that some basal sauropods retained the plesiomorphic cyclical growth patterns. Among the sauropodomorpha it appears that the basal taxa exploited different growth strategies, but the more derived Eusauropoda successfully utilized rapid, uninterrupted growth strategies.

## Introduction

Sauropods, the iconic gigantic dinosaurs of the Mesozoic, reached lengths in excess of 30 m and weights of over 50 tons [1, 2], making them the largest terrestrial vertebrates that ever lived. It is generally held that they achieved gigantism through accelerated growth rates and high basal metabolic rates [2–3]. Sauropods appear to have had high, sustained rates of growth throughout their lives, which only slowed down in late stages of ontogeny with the development of periodic interruptions [2, 3]. This ‘typical’ sauropod growth pattern has been deduced from studies of the microstructure of their long bones, which exhibit an uninterrupted deposition of fibrolamellar bone tissue during the early phase of development, and once sexual maturity is attained, becomes periodically interrupted by growth marks (annuli and/or lines of arrested growth, LAGs) [3]. This kind of growth dynamic has been reported even in the basalmost sampled sauropod dinosaur (i.e., cf. *Isanosaurus* [3]).

Basal sauropodomorphs (i.e. non sauropod sauropodomorphs) are the forerunners of sauropods [4, 5, 6]. Unlike the sauropods, these dinosaurs have periodically interrupted growth during most of their development, evidenced by the formation of fibrolamellar bone interrupted by regularly spaced growth marks [3, 7, 8]. Current hypotheses on the origin of sauropod growth dynamics, however, essentially stem from studies of only a few basal sauropodomorph dinosaur taxa, including *Massospondylus* [8], *Plateosaurus* [7], *Thecodontosaurus* [3, 9], and “*Euskelosaurus*” [10], all of which are considerably distantly related to the derived Sauropoda. The only published data regarding basal sauropods (i.e. non eusauropod sauropods) corresponds with a single sample of a cf. *Isanosaurus* specimen [3]. This dataset therefore also precludes determining when the ‘typical’ sauropod growth pattern originated along the evolution of the Sauropodomorpha, as well as its relationship with the gigantic body size that characterizes sauropods. In this contribution we incorporate critical new data to explore the sauropodomorph growth patterns, in order to evaluate how such growth changes occurred and how they affected the timing of major steps in the evolution of Sauropodomorpha.

**Fig 1:**
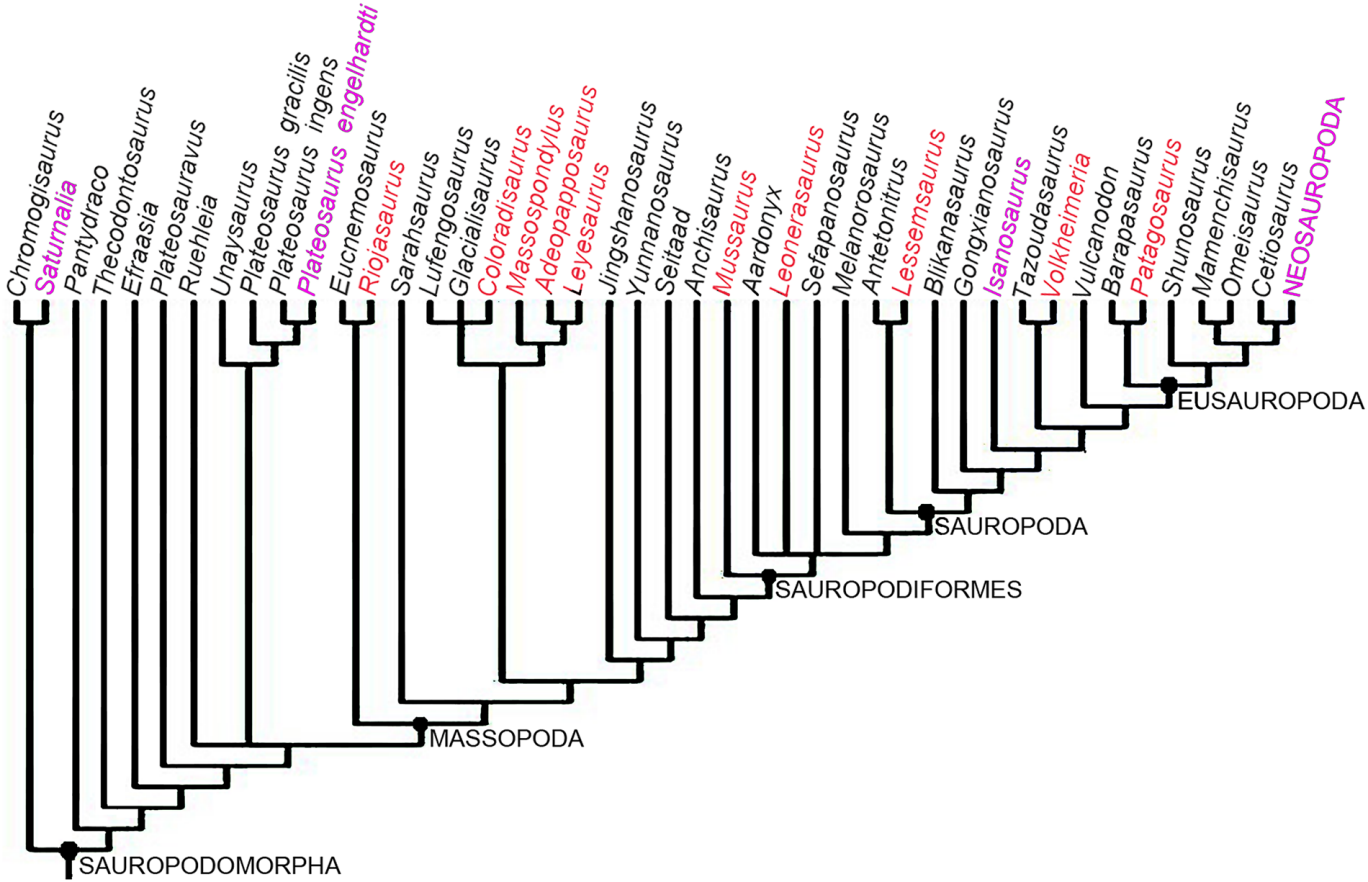
Phylogenetic relationships of basal sauropodomorpha showing the taxa included in this study (red lettering). The tree corresponds with the reduced strict consensus tree from the modified matrix of Otero et al. [40]. Taxa employed for comparison are in magenta lettering. Distinction between stem and node based taxa is not included in the tree.

For this study we examined the femoral histology of seven basal sauropodomorph dinosaur taxa, including forms closely related to Sauropoda (Table 1, Fig 1). We also include three basal sauropod taxa [11, 12], as well as data from published literature on sauropodomorph histology [2, 3, 8, 10, 13–17]. The aims of our study are to expand the knowledge about the growth dynamics of basal sauropodomorphs and to test the hypothesis proposed by Sander et al. [3] concerning the origin of the ‘typical’ sauropod growth strategy.

**Table 1:** Specimens sampled for histological analysis in this study. Total length of and circumference at level of the mid-shaft of the femora are also included. With the only exception of *Riojasaurus* PVL 3669 and *Mussaurus* MLP 61-III-20-22, all the specimens correspond with left femora.

Taxon sampled	Specimen	Locality	Horizon	Age	Length (cm)	Circumference at midshaft (cm)
*Riojasaurus incertus*	PVL 3526	Villa Unión, La Rioja Province, Argentina	Los Colorados Formation	Norian—Rhaetian	47.5	23
	PVL 3669	Villa Unión, La Rioja Province, Argentina	Los Colorados Formation	Norian—Rhaetian	57	23.7
*Coloradisaurus brevis*	PVL 5904	La Esquina, La Rioja Province, Argentina.	Los Colorados Formation	Norian—Rhaetian	53	19.1
*Massospondylus carinatus*	BP/1/4934	Bormansdrift, Clocolan District, Free State Province, South Africa	Elliot Formation	Early Jurassic	55*	20.9**
*Adeopapposaurus mognai*	PVSJ 569	Sierra de Mogna, San Juan province, Argentina	Cañadón del Colorado Formation	Early Jurassic	23.8	7.6
*Leyesaurus marayensis*	PVSJ 1079	Balde de Leyes, San Juan Province, Argentina	Quebrada del Barro Formation	Early Jurassic	39	13.5
*Mussaurus patagonicus*	MLP 61-III-20-22	Laguna Colorada, Santa Cruz Province, Argentina	Laguna Colorada Formation	Late Sinemurian	80	32
	MPM PV 1815	Laguna Colorada, Santa Cruz Province, Argentina	Laguna Colorada Formation	Late Sinemurian	---	22.2
*Leonerasaurus taquetrensis*	MPEF 1663	Cañadón Las Leoneras, Chubut Province, Argentina	Leoneras Formation	Early Jurassic	---	---
*Lessemsaurus sauropoides*	PVL 4822/64	La Esquina, La Rioja Province, Argentina	Los Colorados Formation	Norian—Rhaetian	84	36
*Patagosaurus fariasi*	PVL 4075	Cerro Cóndor, Chubut Province, Argentina	Cañadón Asfalto Formation	Middle Jurassic (Aalenian-earliest Bathonian)	139	61
*Volkheimeria chubutensis*	PVL 4077	Cerro Cóndor, Chubut Province, Argentina	Cañadón Asfalto Formation	Middle Jurassic (Aalenian-earliest Bathonian)	67	25.5

*From [47].

**From [77].

## Materials and methods

Twelve femora from ten sauropodomorph taxa (*Riojasaurus incertus*, *Coloradisaurus brevis*, *Adeopapposaurus mognai*, *Leyesaurus marayensis*, *Massospondylus carinatus*, *Mussaurus patagonicus*, *Leonerasaurus taquetrensis*, *Lessemsaurus sauropoides*, *Volkheimeria chubutensis* and *Patagosaurus fariasi*) were sampled for histological analysis. All the femora belong to different individuals. Data on accession numbers, locality and horizon of these samples are compiled in Table 1. Femoral length and the circumference at the mid-shaft were measured for all the samples. Except for *Mussaurus patagonicus* MPM-PV 1815 and *Leyesaurus marayensis* PVSJ 1079 (both unpublished specimens), all the sampled bones have been confidently assigned to species level in previously published studies [11, 12, 18–24]. Moreover, we include samples obtained directly from holotypic specimens (*Leonerasaurus taquetrensis*, *Volkheimeria chubutensis*) and one neotype (*Massospondylus carinatus*). In the case of *Mussaurus patagonicus* MPM-PV 1815, the taxonomic assignment of this specimen was established based on two autapomorphic features of *M*. *patagonicus* recognized in the astragalus of the specimen (i.e. concave distal lateromedial surface and presence of a well developed convex bulge on the posteromedial margin) [24]. In the case of PVSJ 1079, this specimen is a gracile basal sauropodomorph with massospondylid like features, which is proposed here as a new referred specimen to *Leyesaurus marayensis*. This specimen comes from the same monospecific level of the holotype of *Leyesaurus marayensis* (Balde de Leyes Formation, Marayes-El Carrizal Basin, Northwestern Argentina) [25, 26], which has only provided basal sauropodomorphs that resemble *Leyesaurus*. Other monogeneric sedimentary levels bearing massospondylid sauropodomorphs are known in South America (i.e., *Adeopapposaurus*, from Mogna Basin at Northwestern Argentina). Although PVSJ 1079 and the type specimen of *Leyesaurus* (PVSJ 706) do not share any similar bone elements with autapomorphic features, their overall morphological similarities and the stratigraphic and spatial closeness of both findings (ten meters apart, at the same stratigraphic level), suggest they are the same species. Several anatomical characters are shared between PVSJ 1079, *Leyesaurus*, and/or the rest of massospondylids, such as the presence of a projected posterodorsal corner of middle dorsal neural spines, concave lateral margins of the pubic apron, presence of a well developed brevis fossa with sharp margins on the ventral surface of the postacetabular process of the ilium, pyramidal dorsal process on the posteromedial corner of the astragalus, and a centrally located fourth trochanter along the mediolateral axis of the femur. Most of the *Leonerasaurus* remains were articulated, although a few bones were found within a meter radius of the skeleton. The femur studied here, was found about 30 cm from the articulated sacrum and ilia. Since no other individuals were collected from the quarry, it is highly likely that the femur (which fits the expected size for the rest of the skeleton) belongs to the *Leonerasaurus* holotype.

Specimens were prepared for thin sectioning based on the methodology described in [27, 28]. Samples for thin sectioning where obtained from the midshaft of the femora, preferentially below the fourth trochanter. However, in *Leonerasaurus traquetensis*, the two studied sections where obtained just below and above the fourth trochanter. These thin sections were originally prepared for ontogenetic stage determination in the study of Pol et al. [22]. Since the section taken just above the fourth trochanter did not introduce any major discrepancies in terms of histology, we also included this section in our study. In cases in which a complete element was available for sampling, a 1 cm section was obtained from the bone shaft. To avoid loss of anatomical information, a cast of the extracted sample was generated and used to reconstruct the bone. The histological thin section preparation was carried out in the Museo Paleontológico Egidio Feruglio (Trelew, Argentina), with the exception of *Massospondylus* which was prepared at the University of Cape Town, South Africa, and *Adeopapposaurus*, which was prepared at Instituto y Museo de Ciencias Naturales, Universidad Nacional de San Juan, Argentina. The thin sections were studied using a petrographic polarizing microscope (Nikon E400). Nomenclature and definitions of structures used in this study are derived from [29–31]. In the case of the term “intrinsic fibers”, we follow the definition of [31], for which this term refers to the collagenous fibers that are deposited by osteoblasts during the ossification process and form the matrix of the bone tissue. Although, the actual fibers (like other organic tissue decompose soon after death [30]), the crystallites present in the fossil bone reliably indicate the organization of these “intrinsic fibers”, and for simplicity we refer to these as intrinsic fibers of the bone. The term “modulations” refers to the “cycles” or “depositional cycles” described in some sauropod dinosaurs (e.g. [14]), which consist of cyclical variations in the vascular pattern of the primary bone, which are best observed under low magnifications and does not correspond with distinct growth marks (i.e. lines of arrested growth or *annuli*).

Since osteohistology is affected by ontogenetic age, we estimated the relative ontogenetic stages of each specimen using histology features and external anatomy. For this, we determined the somatic maturity of the specimens (i.e. when adult size is achieved) on the basis of the presence of an outer circumferential layer (OCL), also called external fundamental system (EFS) in the subperiostal layer of the cortex. Such a feature is formed by avascular parallel fibered bone and/or lamellar bone often with abundant closely spaced lines of arrested growth [30]. Following previous studies on nonavian dinosaurs [7, 15, 16, 32–34], we assume that sexual maturity in sauropodomorph dinosaurs occurred well before somatic maturity. According to histological studies on extant vertebrates (e.g. [35, 36]), we inferred sexual maturity in basal sauropodomorph on the basis of a clear change in the organization of the intrinsic fibers (from poorly to well organized) and/or a decreasing in the spacing between successive growth marks. For sauropod dinosaurs, we employ the criterion proposed by Klein and Sander [34], in which the sexual maturity is inferred on the basis of the identification of histologic ontogenetic stages (HOS). According to these authors, thirteen HOS are identified on the basis of a combination of seven different types of bone tissues. Each tissue type is characterized by several features, such as vascularization pattern, degree of secondary remodeling and presence of growth marks [34]. Sexual maturity is reached in HOS 9 or 10. Following Irmis [37], we contrasted all the histological features with anatomical evidence of maturation (i.e. degree of neurocentral closure of the vertebrae), and we utilized the criteria proposed by Brochu [38] for distinguishing between different stages of neurocentral suture closure (see also [37, 39]). We also contrast this information with the relative size of the elements in all sampled specimens (Table 1).

For the study of the evolution of the growth strategies amongst Sauropodomorpha, we optimized two histological characters added to the phylogenetic matrix of Otero et al., [40]. Since the original matrix does not include *Volkheimeria*, we have included this taxon in the phylogenetic dataset for a more complete evaluation of character optimization. In the case of *Isanosaurus*, we scored the histological data based on the specimen sampled by [3], which has been identified as cf. *Isanosaurus*. The original data set was modified by the addition of two histological characters that reflect the growth strategy in the sampled species. The first character, based on the presence of growth marks (LAGs and/or annuli) in the cortical bone, has been treated as a binary character with two different states: growth marks in the whole cortex (0) and growth marks absent or only formed in the outer cortex (1). The second character, also codified as binary, refers to the relative abundance of woven fibered (WFB) or parallel fibered bone (PFB) in the primary compact bone (PFB>WFB:0, WFB>PFB: 1). In the cases in which a single taxon exhibits the two states of the same character (e.g. *Mussaurus*), we codify them as polymorphic (i.e. 0 & 1). Histological data for the scoring has been obtained both from direct observation and from the published literature [2, 3, 9, 15–17, 34, 41, 42]. In the case of *Saturnalia* and *Plateosaurus*, scoring was also based on updated data provided by personal communication with M. Sander—indicating that contrary to what has been published in the literature, the woven fibred component is less than the parallel fibred bone in both of these taxa. In the case of the first character, although other cyclical growth marks have been described for sauropodomorph dinosaurs (e.g. polish lines and modulations), since they do not translate into a real slowing down or arrest of bone growth, we have only considered the presence of annuli and LAGs. Furthermore, to constrain our analysis and avoid intraskeletal histological variation we only considered microstructural descriptions of femora, and to avoid ontogenetic variation, we restricted our analyses to adult or sub adult specimens. The modified matrix was analyzed using TNT v. 1.1 [43, 44]. A heuristic tree search was performed consisting of 1000 replicates of Wagner trees (with random addition sequence of taxa) followed by branch swapping (tree bisection-reconnection; saving ten trees per replicate). The phylogenetic nomenclature used for the clades in this paper is presented in Table 2.

**Table 2:** Phylogenetic nomenclature used in this study.

Clade	Definition	Reference
Sauropodomorpha	The most inclusive clade containing *Saltasaurus* but not *Passer* or *Triceratops*	[53]
Massopoda	The most inclusive clade that contains *Saltasaurus* but not *Plateosaurus*	[78]
Sauropodiformes	The least inclusive clade containing *Mussaurus* and *Saltasaurus*	[53]
Sauropoda	The most inclusive clade containing *Saltasaurus* but not *Melanorosaurus*	[78]
Eusauropoda	The least inclusive clade containing *Shunosaurus* and *Saltasaurus*	[1]
Neosauropoda	*Diplodocus longus*, *Saltasaurus loricatus*, their most recent common ancestor and all its descendants	[79]

### Ethics statement

The specimens involved in this study are publicly deposited and accessible by others in permanent repositories. All specimens described herein or used for comparative purposes are curated in recognized repositories and were accessed with the explicit permission of the appropriate curators and/or collection managers. No permits were required for the described study, which complied with all relevant regulations. Repository locations and abbreviations for all specimens discussed in the text are as follows: BP, Evolutionary Studies Institute, Johannesburg, South Africa (formerly Bernard Price Institute); MLP, Museo de La Plata, La Plata, Argentina; MPEF, Museo Paleontológico ‘Egidio Feruglio’, Trelew, Chubut, Argentina; MPM, Museo Regional Provincial ‘Padre M. J. Molina’, Río Gallegos, Santa Cruz, Argentina; PVL, Instituto ‘Miguel Lillo’, Tucumán, Argentina; PVSJ, Paleontología de Vertebrados—Instituto y Museo de Ciencias Naturales, Universidad Nacional de San Juan, San Juan, Argentina.

### Histological description

#### Synopsis of results

Table 3 provides a summary of the main histological features of the taxa examined in this study. An OCL is only present in one of the sampled specimens of *Riojasaurus* (PVL 3526). The basal sauropodomorph dinosaurs have a predominance of parallel fibered bone tissue. An exception to this pattern was encountered in a specimen of *Mussaurus*, which exhibits abundant fibrolamellar bone (i.e. matrix of woven fibered bone in which the vascular spaces are filled with lamellar bone) in the compacta. In the three basal sauropods, *Lessemsaurus*, *Patagosaurus* and *Volkheimeria*, woven fibered bone (i.e. with its mass monorefringence and disorganized globular osteocyte lacunae [45]) is abundant in the compacta.

**Table 3:** General histological features of the studied taxa.

Specimen	Matrix	Vascularization	Growth marks			Marrow cavity	Special features
			Inner cortex	Mid cortex	Outer cortex		
*R*. *incertus* PVL 3526	PFB>>WFB	C>>R≈L	present	present	present	free	Abrupt change of the vascularization pattern at the outer cortex
*R*. *incertus* PVL 3669	PFB>>WFB	C>>R≈L	-----	present	present	free	Vascularization becomes plexiform/reticular in some areas
*C*. *brevis* PVL 5904	PFB>>WFB	C>>R≈L	present	present	present	free	----
*A*. *mognai* PVSJ 569	PFB>>>WFB	C>>R>L	present	present	present	free	----
*L*. *marayensis* PVSJ 1079	PFB>WFB	L>C≈R	present	present	present	free	High secondary remodelation. Concentric rows of secondary osteons
*M*. *carinatus* BP/1/4934	PFB>WFB	L>>C≈R	present	present	present	free	----
*M*. *patagonicus* MLP 61-III-20-22	WFB>>PFB	L>C≈R	absent	absent	present	free	----
*M*. *patagonicus* MPM-PV 1815	PFB>WFB	C>L≈R	absent	absent	present	free	----
*L*. *taquetrensis* MPEF 1663	PFB>>WFB	L≈C	present	present	----	free	----
*L*. *sauropoides* PVL 4822/64	WFB>>PFB	RET/PL	present	present	present	free	Thick zones of reticular to plexiform/fibrolamellar bone
*V*. *chubutensis* PVL 4077	WFB	L≈C>>R	absent	absent	absent	cancellous	Abrupt change of the vascularization pattern at the outer cortex
*P*. *fariasi* PVL 4075	WFB	L≈C>R	absent	absent	present	cancellous	----

Abbreviations: C: circumferential canals; L: longitudinal canals; PFB: parallel fibered bone; PL: plexiform pattern; R: radial canals; RE: reticular pattern; WFB: woven fibered bone.

Generally in the most basal sauropodomorph dinosaurs, growth marks occur throughout the cortex, suggesting periodic slow down or change in the rate of growth throughout ontogeny. However, in *Mussaurus* the growth marks begin only in the outer third of the cortex. It is curious that the bone tissue in the three basal sauropod taxa were not equivalent to one another: *Lessemsaurus* exhibits well defined growth marks throughout the cortical bone, whereas in *Volkheimeria*, although typical growth marks do not occur, poorly defined modulations in the vascularization pattern (i.e. alternating changes between longitudinal to more circumferential canals) are visible in the cortex. Finally in *Patagosaurus*, distinct though poorly formed annuli (with slightly more organized intrinsic fibers and less vascularization) occur throughout the compacta, and are best developed toward the outer cortex.

#### Detailed histology

**Fig 2:**
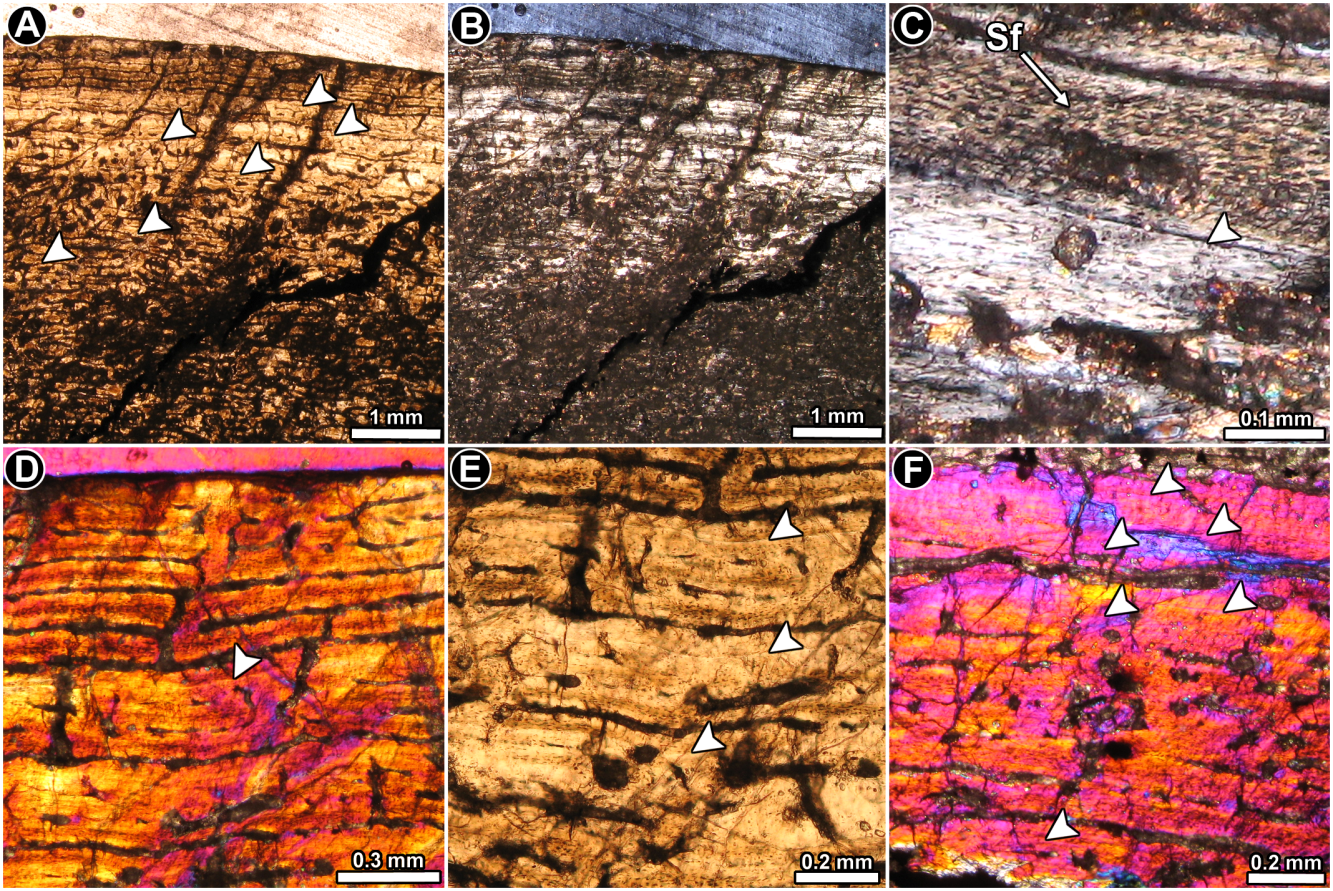
Femur histology of *Riojasaurus incertus* PVL 3526. (**A, B**) General view of the external cortex in normal transmitted (**A**) and polarized light (**B**). Note the predominance of well organized intrinsic fibers in the unaltered portions of the sample (bright areas of the outer cortex in B). Arrowheads indicate the presence of LAGs. (**C**) Detail of the primary bone tissue showing well organized intrinsic fibers and flattened osteocyte lacunae. A growth line (arrowhead) and several Sharpey’s fibers are also observed. Polarized light. (**D**) Enlarged view of the outer cortex showing a distinctive variation in the vascularization pattern in the compact bone. Such variation starts just above the last preserved LAG (arrowhead). Polarized light with lambda compensator. (**E**) LAGs (arrowheads) formed in the outer cortex. Normal transmitted light. (**F**) detailed view of the last six LAGs (arrowheads) formed in the outer cortex. Note the scarce vascularization of the outermost region. Polarized light with lambda compensator. Abbreviations: Sf: Sharpey’s fibers.

*Riojasaurus incertus* PVL 3526: The histology of the bone is not ideally preserved, however, the main microstructural features are discernable in some areas. The cortical bone is mostly composed of highly vascularized primary bone tissue (Fig 2A). Intrinsic fibers are mostly well organized, forming a typical matrix of parallel fibered bone tissue (Fig 2B). Osteocyte lacunae are flattened and they are oriented following the intrinsic fiber arrangement [45] (Fig 2C). Abundant Sharpey’s fibers occur in the anterior portion of the cortex (Fig 2C). Vascularization is extensive and mostly consists of long circumferentially oriented canals, although local variations are evident. For example, toward the posterior region, circumferential vascular spaces become shorter and they anastomose with abundant radial and oblique canals. The outermost layer of bone tissue is poorly vascularized. A particular histological variation is observed in the outer preserved area of the medial region of the cortex. In this region, the bone tissue formed after the last preserved growth mark exhibits relatively more circumferential canals, which tend to extend longer than those observed below the growth mark (Fig 2D). The vascular spaces are also relatively larger and more densely distributed than in the subjacent tissue. Lines of arrested growth are formed in the whole compacta (Fig 2A, 2E and 2F), but their number could not be estimated due to the poor histological preservation of the sample. The anterior outermost cortex exhibits an almost avascular layer of parallel fibered bone, which possesses at least six closely spaced LAGs (Fig 2F). We interpret this structure as an OCL. Resorption cavities and secondary osteons are observed around the perimedullary cavity, particularly in the medial region, where they reach the mid cortex.

**Fig 3:**
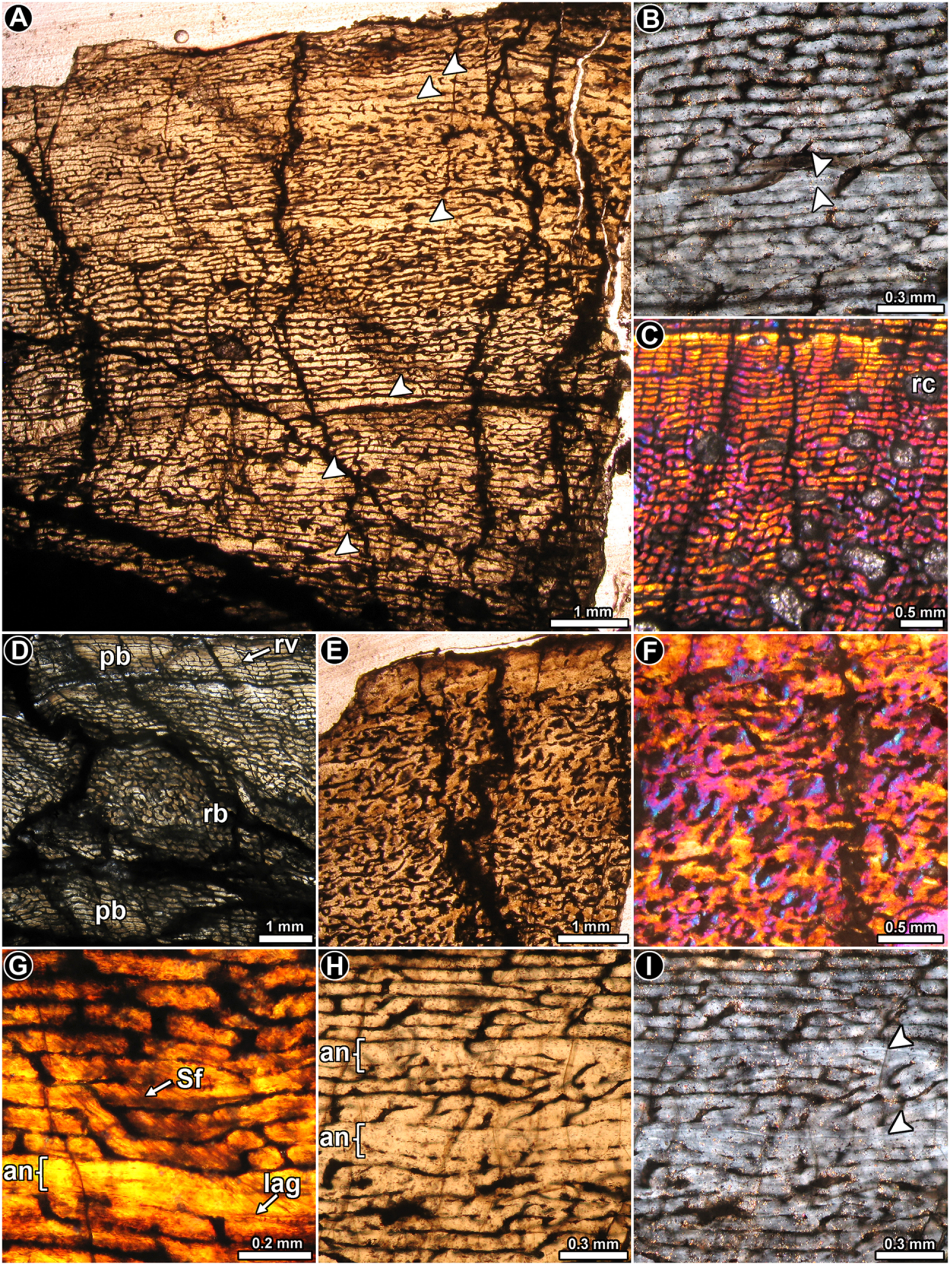
Femur histology of *Riojasaurus incertus* PVL 3669. (**A**) General view of the cortical bone. Growth marks (arrowheads) are formed from the inner to the outer region of the compacta. Normal transmitted light. (**B**) Detail of the primary bone, which is composed of parallel fibered bone (see mass birefringence). Arrowheads indicate a double LAG. Polarized light. (**C**) Highly vascularized woven fibered bone in the inner cortex. Note the variation of the vascularization pattern from the inner to the outer area. Polarized light with lambda compensator. (**D**) General view of the inner cortex showing the variation of the vascular canals arrangement (plexiform in the inner region, reticular in the middle and plexiform again in the outer region). Normal transmitted light. (**E, F**) General (**E**) and detailed (**F**) view of the anterior portion of the cortex showing the irregular arrangement of the vascular canals. Normal transmitted (**E**) and polarized light with a lambda compensator (**F**). (**G**) Detail of Sharpey’s fibers. A single LAG accompanied by an annulus is also observed in the figure. (**H, I**) Detailed view of LAGs (arrowheads) and annuli in the cortical bone viewed under normal transmitted (**H**) and polarized light (**I**). Abbreviations: an: annulus; lag: line of arrested growth; pb: plexiform bone; Sf: Sharpey’s fibers; rb: reticular bone; rv: radially oriented vascular canal; rc: resorption cavity.

*Riojasaurus incertus* PVL 3669: A distinctive expansion of the medullary cavity is evident in this specimen due to diagenesis. However, although only a small part of the cortical bone has been preserved in this specimen as compared to PVL 3526, its fine microstructure is better preserved. The cortical bone is composed almost entirely of highly vascularized primary bone tissue (Fig 3A). In general, the degree of organization of the intrinsic fibers of the matrix varies from moderate (slight birefringence) to high (mass birefringence) (Fig 3B). When preserved, osteocyte lacunae are mostly flattened and well organized. Local variations in the intrinsic fiber arrangement are observed in the posteromedial and lateral regions of the inner cortex. In these areas, the matrix does not exhibit a mass birefringence and consist of woven fibered bone (Fig 3C). Long circumferential canals predominate in the compacta (some of them enlarged due to diagenesis), giving this tissue a rather laminar appearance. Radial canals are frequent, but their density is highly variable around the cortex. Distinctive variations in the vascularization pattern are observed in the inner lateral, inner posteromedial and in the complete anterior portions of the cortex: the vascular pattern in the inner and mid lateral region changes from laminar to reticular, while in the inner portion of the posteromedial cortex, a band of plexiform to reticular bone is “sandwiched” between laminar/subplexiform tissue (Fig 3D). Finally, the vascular canals located at the anterior cortex exhibit an irregular arrangement (Fig 3E and 3F). All these changes in the vascularization pattern are accompanied by changes in the intrinsic fiber organization of the matrix. Besides the important variation in the shape of the vascular spaces and their arrangement, the anterior cortex is also characterized by the presence of abundant, dense Sharpey’s fibers, which are oriented perpendicularly or slightly oblique to the outer surface. These extrinsic fibers extend from the inner to the outer cortex, and they are so abundant that they tend to obscure the arrangement of the intrinsic fibers in the region (Fig 3F). Sharpey’s fibers are also present in the medial cortex, but they are not as abundant as in the anterior region, and they are oriented more obliquely to the outer surface (Fig 3G). Cyclical growth marks are expressed as annuli and LAGs (Fig 3A, 3B and 3G–3I). Annuli are characterized by a high organization of their fibers (strong birefringence) and a low degree of vascularization. LAGs (single or double) are recognized as thin birefringent lines in the matrix. At least seven growth marks relatively equidistant from one another are recognized in the compacta. Except for the anterior quadrant of the cortex, resorption cavities mostly occur in the perimedullary region, particularly in the posterior area. Some notably large resorption cavities are present in the inner medial cortex, and they are more or less concentrically arranged. A few remnants of secondary osteons are discernable in the fragments of compact bone within the medullary cavity.

**Fig 4:**
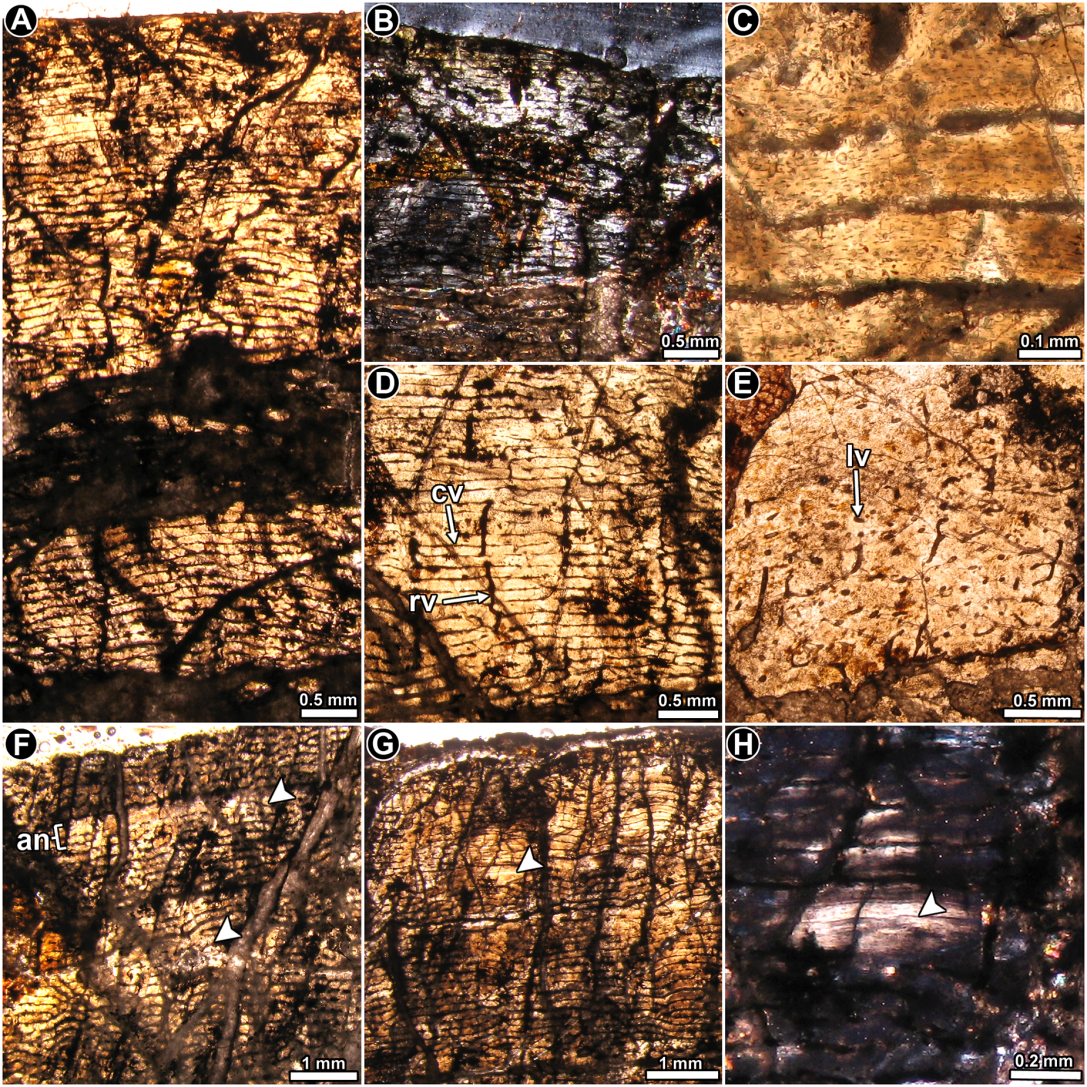
Femur histology of *Coloradisaurus brevis* PVL 5904. (**A**) General view of the cortex, which is composed almost entirely of well vascularized primary bone tissue. Normal light. (**B**) General view of the cortical bone showing the predominance of parallel fibered bone in the compacta. Polarized light. (**C**) Enlarged view of the primary bone. Note the flattened appearance of the osteocyte lacunae and its circumferential arrangement. Normal transmitted light. (**D**) Primary bone tissue in which circumferential vascular canals predominate, and are often anastomosed by large radial canals. Normal transmitted light. (**E**) Primary bone tissue in which longitudinally oriented vascular canals predominate, with some anastomosing oblique canals. Normal transmitted light. (**F**, **G**) General view of the cortex showing a stratified pattern in the primary bone tissue caused by the presence of poorly vascularized annuli (arrowheads). Normal transmitted light. (**H**) Detailed view of a LAG in mid cortex. Polarized light. Abbreviations: an: annulus; cv: circumferentially oriented vascular canal; lv: longitudinally oriented vascular canal; rv: radially oriented vascular canal.

*Coloradisaurus brevis* PVL 5904: The thin section shows that extensive diagenetic alteration occurred, which precludes a complete histological assessment. From the parts that are reasonably well preserved, it is evident that the cortex consists of well vascularized primary bone (Fig 4A), in which most of the matrix exhibits parallel fibered bone with its characteristic mass birefringence, and flattened osteocyte lacunae arranged in parallel (Fig 3B and 3C). Local variations of the intrinsic fiber arrangement are visible in some areas (e.g. anterior region), in which the matrix grades into a woven bone tissue. Intermediate conditions between typical parallel and woven fibered bone are also recognized. Vascular canals are abundant and are organized as primary osteons. These structures are mainly circumferentially arranged in concentric laminae, which anastomose with radial or oblique canals (Fig 4D). Since the density of radial and oblique canals is variable in the compacta, the vascularization pattern varies from a laminar to a plexiform condition. Regional variation is observed in the anterior cortex, where the primary osteons are not formed in laminae and are mostly longitudinally oriented, with several oblique anastomoses (Fig 4E). Variation in the vascularization pattern is also observed in the inner area of the posteromedial cortex, in which the pattern becomes more reticular. Vascularization does not appear to decrease toward the periosteal surface.

The primary matrix is interrupted by concentric growth marks (LAGs and annuli). Due to the poor preservation of the sample, it is not possible to establish the total number of growth cycles. Annuli are easily recognized since they show a significant reduction in vascularization (Fig 4F–4H). When preserved, LAGs are observed as thin, bright lines. A reduction of the space between successive growth marks is not evident in the sample. Resorption cavities are commonly formed in the inner portion of the anterior and posterolateral cortices. In the anterior region, these spaces (and some secondary osteons in early stages of development) are present in the outer cortex.

**Fig 5:**
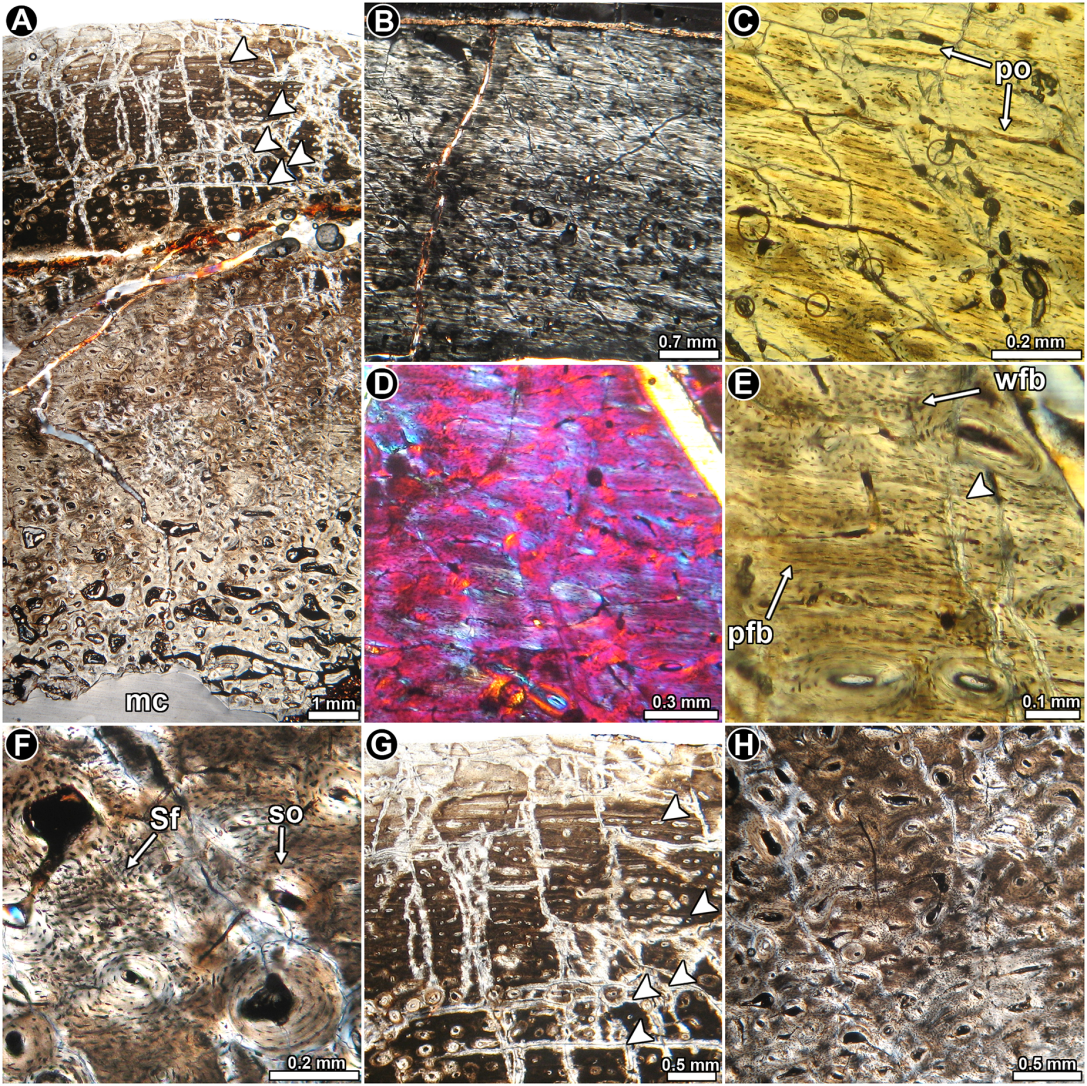
Femur histology of *Massospondylus carinatus* BP/1/4934. (**A**) General view of the compact bone. Note the extensive secondary remodeling in the inner half of the compacta. Several LAGs are observed in the primary bone tissue (arrowheads). Normal light. (**B**) Primary bone tissue at the outer cortex. Some degree of birefringence is evident in the matrix. Polarized light. (**C**) Detailed view of the primary bone tissue showing primary osteons and strongly flattened osteocyte lacunae. Normal light. (**D**) Detail of the mid cortex showing predominance of fibrolamellar bone tissue. Polarized light with lambda compensator. (**E**) Enlarged view of the primary bone showing variation in the shape of the osteocyte lacunae shape and their arrangement. The bone matrix is interrupted by a single LAG. Normal light. (**F**) Detail of secondary osteons and Sharpey’s fibers. Normal light. (**G**) Primary bone at outer cortex interrupted by LAGs. Normal light. (**H**) Detailed view of the inner cortex showing abundant secondary osteons. Normal light. Abbreviations: pfb: parallel fibered bone; po: primary osteons; Sf: Sharpey’s fibers; so: secondary osteons; wfb: woven fibered bone.

*Massospondylus carinatus* BP/1/4934: The thin section comprises of only a part of the diaphysis, and shows some diagenetic alteration (Fig 5A). The arrangement of the intrinsic fibers of the primary bone is variable. In several areas, the matrix exhibits an intermediate condition between typical parallel and woven fibered bone tissue, showing at least some degree of extinction under polarized light (Fig 5B). It is also accompanied by elongated osteocyte lacunae arranged in parallel (Fig 5B). However, distinctive woven fibered bone is also present in the sample, particularly toward the inner cortex (Fig 5D and 5E). Longitudinally oriented primary osteons are commonly arranged in concentric laminae. In an area of the cortex, the organization of the vascular spaces is more irregular, and longitudinally arranged osteons predominate. The density of vascular spaces decreases substantially in the outer cortex. Sharpey’s fibers are well formed in some areas of the cortex (Fig 5F), and they are particularly abundant in the region in which the vascular spaces become more irregularly arranged. The cortex exhibits a clear pattern of stratification, which is produced by the presence of LAGs and, in some cases, annuli of parallel fibered bone (Fig 5A and 5G). The poor preservation of the overall cortex makes a count of the growth marks difficult, but there appears to be about 14 growth marks.

Resorption cavities and secondary osteons are quite abundant in the perimedullary region of the cortex. The resorption cavities show irregular shapes and they usually coalesce to form larger spaces. Secondary osteons are also present in the mid cortex, and are more abundant in some areas of the cortex (Fig 5H). In some places they are organized concentrically.

**Fig 6:**
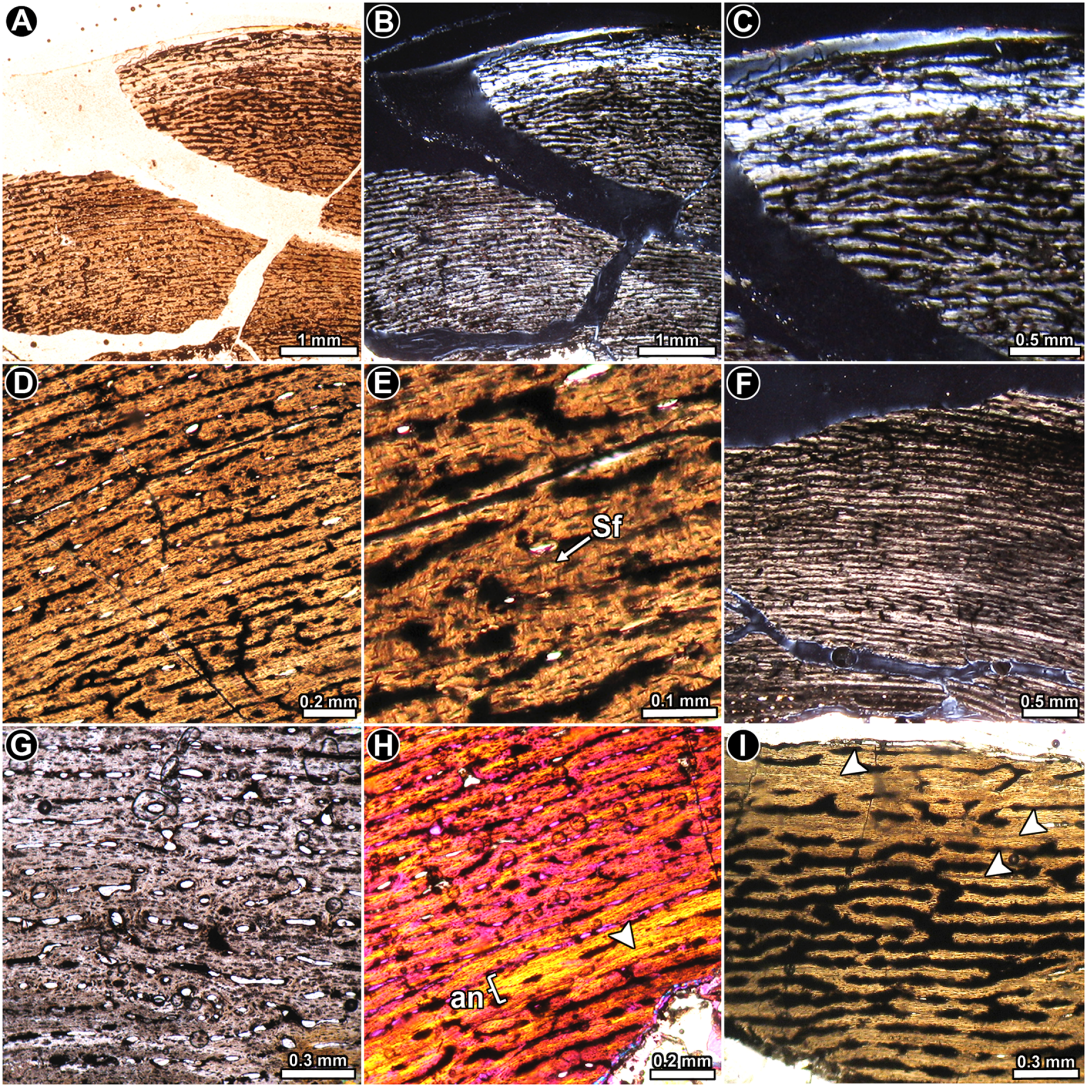
Femur histology of *Adeopapposaurus mognai* PVSJ 569. (**A**, **B**) General view of the cortex in normal transmitted (**A**) and polarized light (**B**). Note that intrinsic fibers exhibit a moderate to high degree of organization. (**C**) Detailed view of the outer cortex showing the high degree of birefringence at the subperiosteal region of the compacta. Polarized light. (**D**, **E**) General view (**D**) and detail (**E**) of the primary bone tissue. Note the Sharpey’s fibers embedded in the tissue. Normal light. (**F**) General view of the cortical bone showing the predominance of circumferentially arranged vascular canals. Polarized light. (**G**) Detailed view of the cortex showing the predominance of longitudinally oriented vascular canals in this area. Normal light. (**H**) Poorly defined annulus and LAG in the inner cortex (arrowhead). Polarized light with lambda compensator. (**I**) Detail of the outer cortex showing three LAGs (arrowheads). Normal light. Abbreviations: an: annulus; Sf: Sharpey’s fibers.

*Adeopapposaurus mognai* PVSJ 569: The cortex is composed predominantly of highly vascularized primary bone tissue (Fig 6A and 6B). The osseous matrix mainly consists of parallel fibered bone, in which the organization of the intrinsic fibers range from slight to well organized (Fig 6B). Some fragments (e.g. isolated splinters of bone located inside the medullary cavity near the inner region of the anterior cortex) have woven fibered bone. The highest extent of fiber organization is observed in the outermost cortex, in which the matrix exhibits a strong birefringence (Fig 6B and 6C). The osteocyte lacunae have an elongated shape and they are commonly highly organized (Fig 6D and 6E). Branching canaliculi are well preserved in some areas of the cortex. Sharpey’s fibers are recognized in the anterior region, extending from the inner to the outer portions of the cortex (Fig 6E).

Vascularization is extensive throughout the compacta, with circumferentially oriented canals predominating (Fig 6F). These relatively short canals often anastomose with oblique ones. Longitudinally oriented canals become more abundant in the anterior and posteromedial areas of the cortex (Fig 6G). There is no evidence of a reduction in the degree of vascularization toward the outer surface.

Growth marks are not distinctive, although there is a narrow band in the inner cortex in which the fibers exhibit a higher degree of organization. This band, accompanied by a poorly defined LAG, could be interpreted as an annulus (Fig 6F and 6H). Three other LAGs are observed in the outer cortex (Fig 6I).

**Fig 7:**
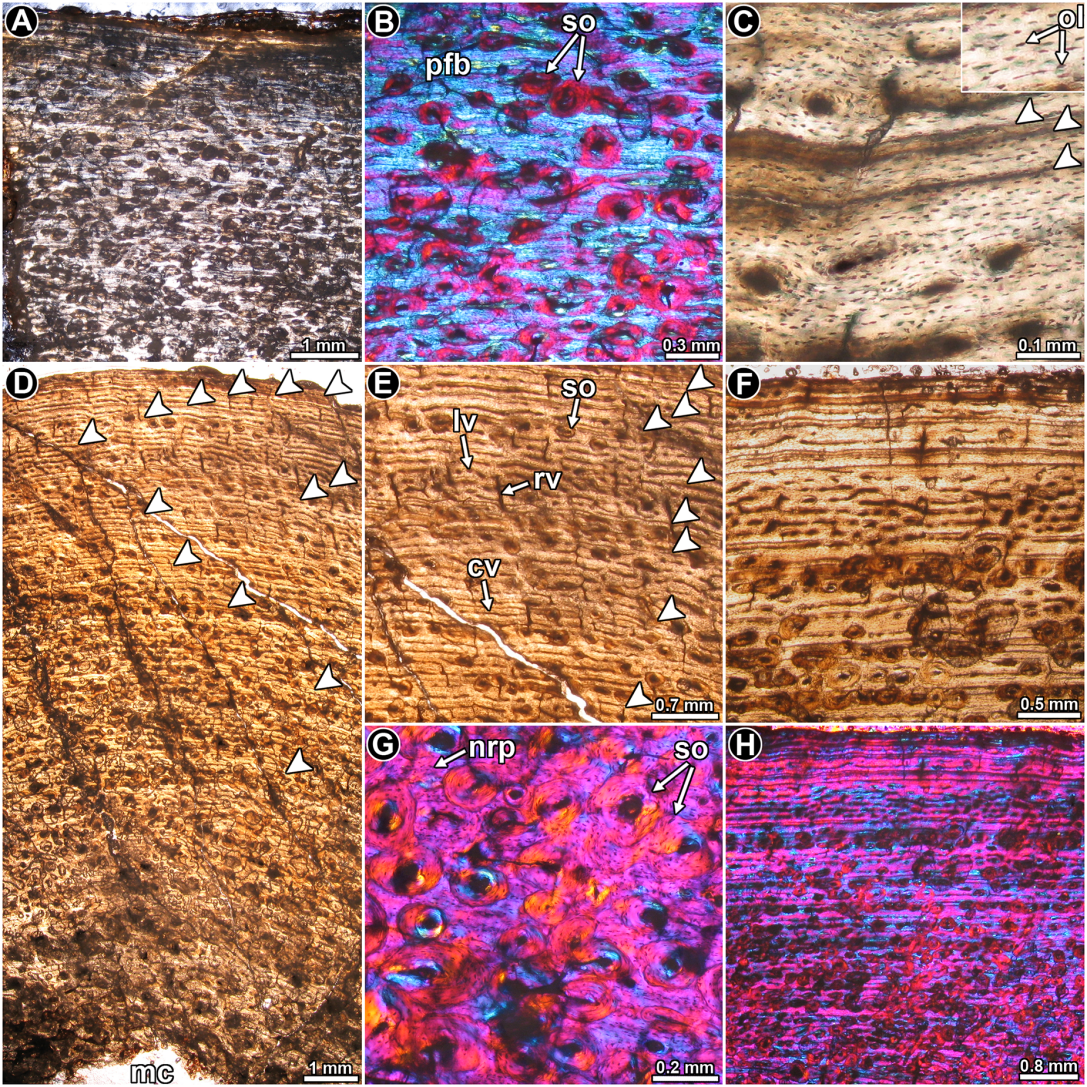
Femur histology of *Leyesaurus marayensis* PVSJ 1079. (**A**, **B**) General view (**A**) and detail (**B**) of the cortical bone. Note the mass birefringence of the primary bone tissue. Polarized light (**A**) and polarized light with lambda compensator (**B**). (**C**) Detailed view of the primary bone tissue showing flattened osteocyte lacunae. Double and single LAGs are also observed. Normal transmitted light. (**D**, **E**) General view (**D**) and detail (**E**) of the cortex showing the arrangement of vascular canals and the relative distribution of LAGs. Normal transmitted light. (**F**) Enlarged view of the outer cortex. Note that the spacing between successive LAGs is reduced toward the periosteal surface. Normal transmitted light. (**G**) Highly remodeled portion of the cortex showing different generations of secondary osteons. Despite the relative abundance of the secondary osteons, interstitial primary bone is preserved. Polarized light with lambda compensator. (**H**) General view of the compact bone showing abundant secondary osteons, which are arranged in concentric rows in some areas. Polarized light with lambda compensator. Abbreviations: cv: circumferentially oriented vascular canal; nrp: non remodeled primary bone tissue; lv: longitudinally oriented vascular canal; mc: medullary cavity; ol: osteocyte lacunae; pfb: parallel fibered bone; rv: radially oriented vascular canal; so: secondary osteons.

*Leyesaurus marayensis* PVSJ 1079: The intrinsic fibers of the bone matrix vary between the typical disorganized arrangement of woven bone to the more organized patterns observed in parallel fibered bone, with the latter being more prevalent (Fig 7A and 7B). In other words, intrinsic fiber organization in the bone mostly grades from slightly to well ordered in the compacta. Osteocyte lacunae have rounded to flattened shapes, and their organization mirrors the arrangement of the intrinsic fibers in the matrix (Fig 7C). The compacta is highly vascularized with primary osteons that are mostly longitudinally oriented. Circumferentially arranged canals are also common. These circumferentially oriented vascular spaces are regularly anastomosed with radial and oblique canals, giving a more plexiform appearance to the tissue (Fig 7D). As occurs in other samples, the vascularization pattern is not homogeneous, and local variation is evident. For example, whereas at the posterolateral region the canals are mostly longitudinally oriented, they become more circumferentially arranged in the medial region of the compacta. This variation is not only observed between different portions of the section, but also at different parts of the compacta in the same region. Distinct LAGs (single, double and even triple) are clearly observed in the whole cortex (Fig 7C–7F). A total of thirteen LAGs are observed in the sample. The lines formed in the outer cortex are clearly more closely spaced (Fig 7F). Despite the closeness of these LAGs, the zones between them show vascular spaces.

Secondary osteons are well developed in the compacta (Fig 7G). They are observed from about the inner 2/3 of the bone wall. The outer cortex contains mainly isolated secondary osteons, but in the inner cortex they tend to be grouped together, although they never form dense Haversian bone. In several instances, secondary osteons are organized in concentric rows (Fig 7H). These rows commonly coincide with growth marks. The secondary osteons tend to be longitudinally oriented, but oblique canals are also often formed in the perimedullary cortex.

**Fig 8:**
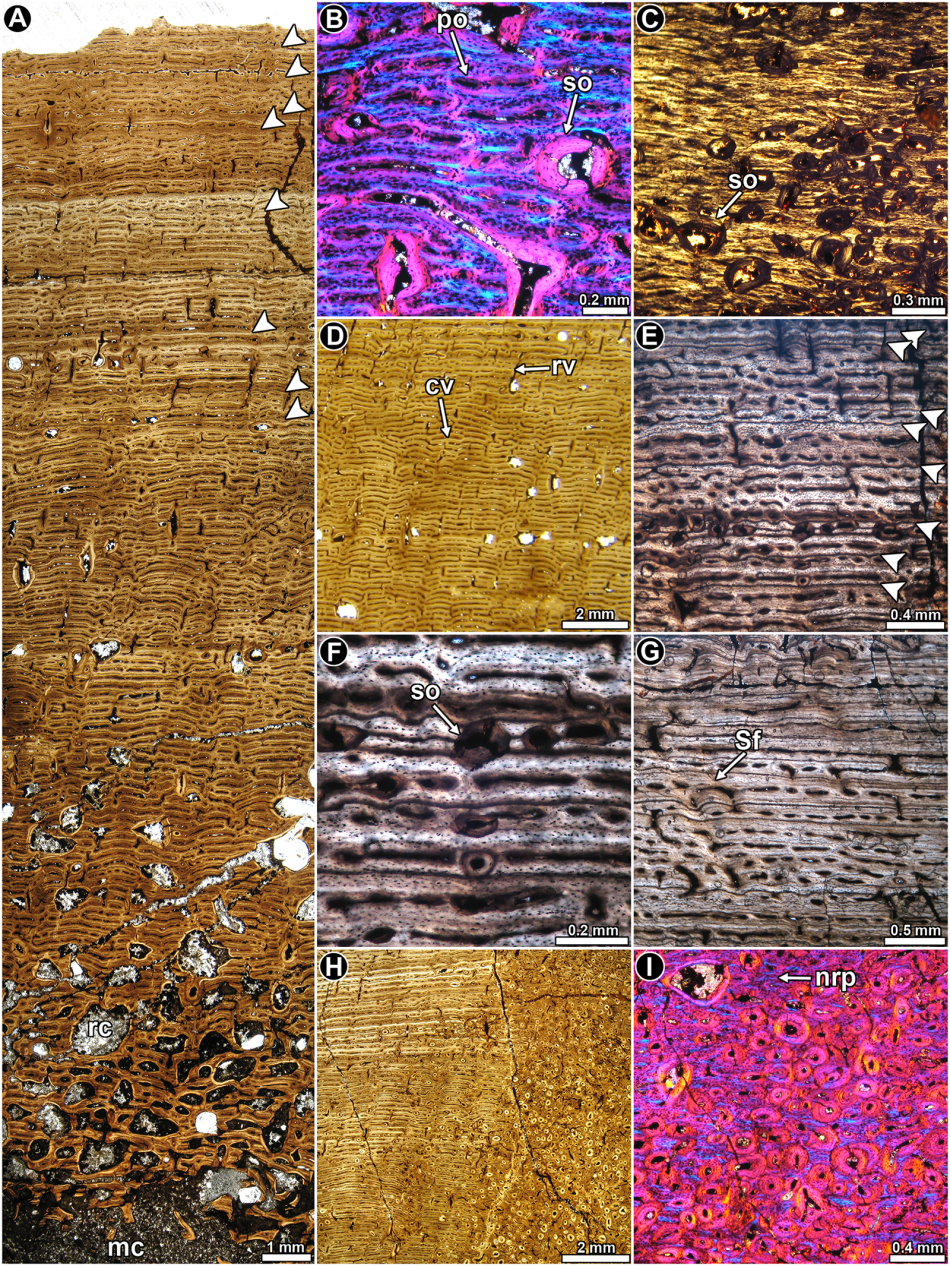
Femur histology of *Mussaurus patagonicus* MLP 61-III-20-22. (**A**) General view of the compact bone. Arrowheads indicate the presence of LAGs in the outer third of the compacta. Normal light. (**B**) Detailed view of the fibrolamellar bone tissue. Primary bone is interrupted by some secondary osteons. Polarized light with lambda compensator. (**C**) Detail of the cortical bone showing predominance of parallel fibered bone tissue. Polarized light. (**D**) Detail of the highly vascualrized primary bone. Vascular canals are exhibits a plexiform pattern. Normal light. (**E**) Detailed view of the outer cortex showing several LAGs (arrowheads). Note that some secondary osteons are organized in concentric rows. Normal light. (**F**) Detailed view of the primary bone showing closely spaced LAGs and secondary osteons. (**G**) Closely spaced LAGs at the outer lateral region. Note the presence of some bundles of Sharpey’s fibers. Normal light. (**H**) Enlarged view of the cortex showing the presence of abundant secondary osteons developed only in the anterolateral region of the cortex (right half of the image). Normal light. (**I**) Detail of the most remodeled area of the compacta. Note that interstitial primary bone tissue is still preserved. Polarized light with lambda compensator. Abbreviations: cv: circumferentially oriented vascular canal; mc: medullary cavity; nrp: non remodeled primary bone tissue; po: primary osteon; rc: resorption cavity; rv: radially oriented vascular canal; Sf: Sharpey’s fibers; so: secondary osteon.

*Mussaurus patagonicus* MLP 61-III-20-22: The thin section of the femoral midshaft is composed of a thick layer of compact bone, which surrounds a large free medullary cavity (Fig 8A). The cortical bone tissue consists predominantly of highly vascularized fibrolamellar bone (Fig 8B). The fibrous matrix of the fibrolamellar tissue is not regularly woven and, under polarized light, several regions (e.g. anterior region and outer cortex) of the bone appear to be well organized (showing general anisotropy under crossed nicols) (Fig 8C). Vascular canals tend to mostly have a plexiform arrangement (Fig 8D), although in some regions longitudinally oriented canals predominate. In other areas, instead, a circumferential arrangement of the canals is present. At least eight lines of arrested growth are observed in the compacta, but these are restricted to the outer third of the cortex. These growth marks tend to be more closely spaced toward the external part of the cortex (Fig 8E–8G). Closely spaced growth marks indicative of substantial truncation in growth appears to be preserved in the lateral region of the cross section (Fig 8G). However, given that the most external part of the cortex has been eroded, it is not possible to confirm that this reflects a cessation of growth. Sharpey’s fibers, penetrating the cortical bone at straight or slightly oblique angles are especially abundant in the outer cortex at the anterolateral region of the cross section. A pathological tissue occurs in the perimedullary region of the sample and this has been previously described [46].

Secondary osteons are commonly developed in the mid to perimedullary region of the cortex. In the anteromedial and anterolateral regions of the cross section, secondary osteons form two wide “columns” of dense Haversian bone (Fig 8H and 8I). In some areas (e.g. lateral region of the cortex), some LAGs are interrupted by laminae of secondary osteons (Fig 8E). Large resorption cavities are present in the perimedullary region (Fig 8A), and these tend to be larger in the posterior and anterior regions of the cortex. The erosion spaces located at the innermost cortex tend to be lined by thin centripetal deposits of lamellar bone.

**Fig 9:**
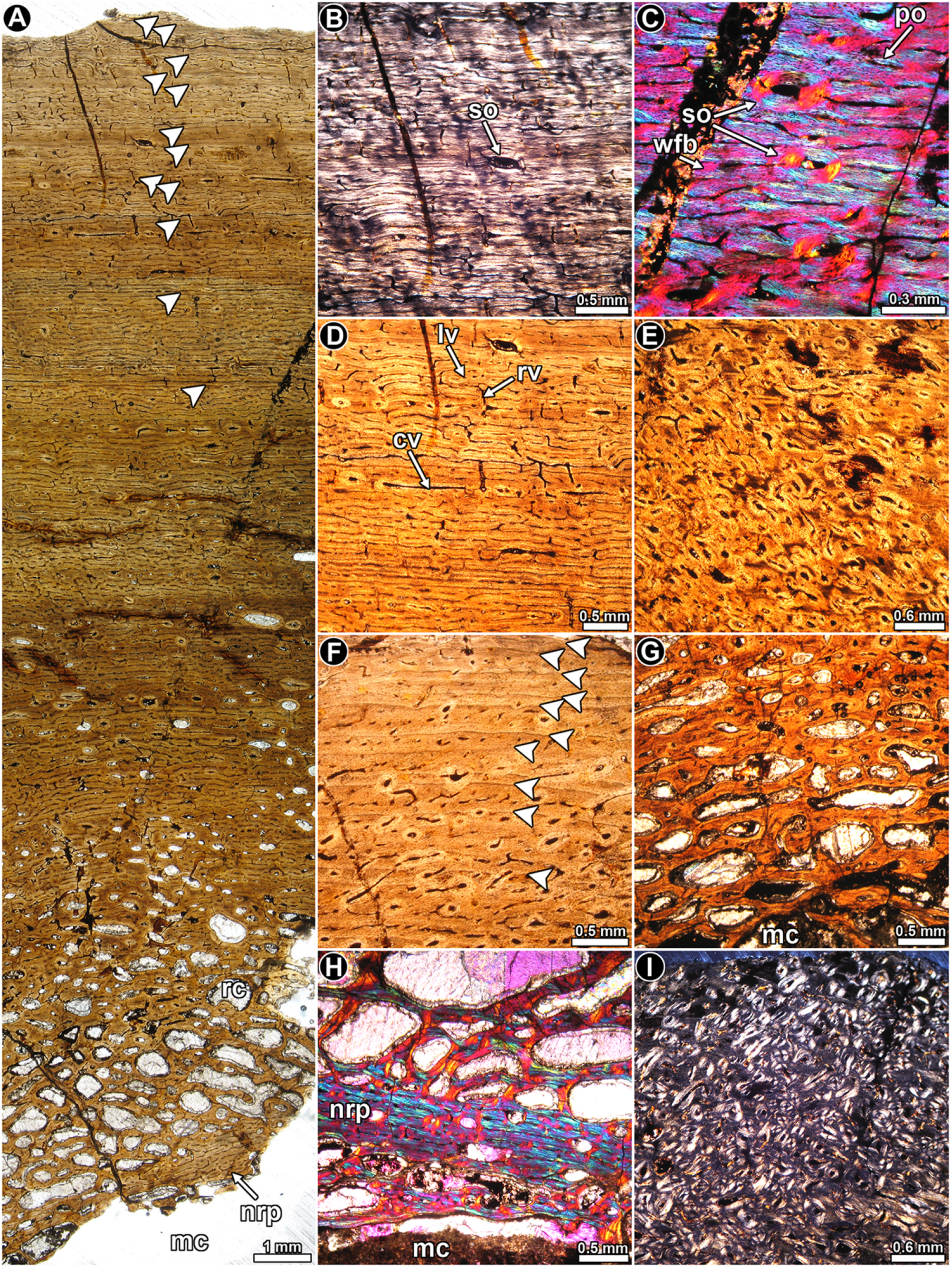
Femur histology of *Mussaurus patagonicus* MPM-PV 1815. (**A**) General view of the compact bone. Arrowheads indicate the presence of LAGs in the outer third of the compacta. Normal transmitted light. (**B**) Detailed view of the outer cortex showing predominance of parallel fibered bone tissue. Polarized light. (**C**) Detail of the fibrolamellar bone tissue in the mid cortex. Polarized light with lambda compensator. (**D**) Primary bone tissue showing a plexiform pattern of vascularization, with a predominance of circumferential canals. Normal transmitted light. (**E**) Detail of the anteromedial cortex showing abundant longitudinally oriented vascular canals, which are anastomosed by oblique canals. Note the presence of several secondary osteons. Normal transmitted light. (**F**) Closely spaced LAGs in the outer cortex. Normal transmitted light. (**G**) Detail of the inner cortex showing abundant large resorption cavities. Normal transmitted light. (**H**) Detail of the inner cortex showing abundant resorption cavities. Note the presence of a distinctive layer of primary bone in the innermost region. Polarized light with lambda compensator. (**I**) Detailed view of the outer cortex showing abundant secondary osteons. Polarized light. Abbreviations: cv: circumferentially oriented vascular canal; lv: longitudinally oriented vascular canal; mc: medullary cavity; nrp: non remodeled primary bone tissue; rc: resorption cavity; rv: radially oriented vascular canal; so: secondary osteons; wfb: woven fibered bone.

*Mussaurus patagonicus* MPM-PV 1815: Three transverse sections were obtained from this specimen, but they are described together since they have similar histological features. The cortical bone is mostly primary in origin (Fig 9A). Parallel fibered bone predominates in the entire cortex, with its birefringence grading from moderate (in the inner to mid cortex) to strong (in the outer cortex) (Fig 9B). The intrinsic organization of the fibers of the matrix becomes more disorganized (woven fibered) in the posteromedial region of the cortex, where the bone tissue is mostly monorefringent (Fig 9C). Except in the outermost cortex, where the vascularization decreases, vascular spaces are extensively developed throughout the compacta. The arrangement of the vascular canals is mostly plexiform with a predominance of circumferential canals (Fig 9D). In the outer cortex the vascular canals are mainly longitudinally oriented. Given that the amount of lamellar bone around the vascular canals is scarce or even nonexistent, primary osteons are poorly developed. An irregular orientation of the primary osteons is visible at the anteromedial and anterolateral regions of the compacta (Fig 9E). Such a change coincides with the presence of Sharpey’s fibers in the cortex. These fibers are densely packed and with respect to the outer surface they are oriented perpendicularly (in the anteromedial region) or obliquely (in the anterolateral region). A minimum of twelve LAGs (including double LAGs), were counted in the outer third of the cortex (Fig 9A). The LAGs are more closely spaced in the outer cortex, where they are accompanied by some annuli consisting of avascular parallel fibered bone (Fig 9F).

The presence of abundant resorption cavities in the perimedullary region gives this area a rather cancellous appearance (Fig 9G). The density and relative size of the resorption cavities varies around the medullary cavity. For example, these spaces are less abundant in the anteromedial region, and they are larger in the medial side than in the anterolateral side. In some areas (e.g. lateral region), concentric rows of large resorption cavities are visible. The resorption process does not appear to progress in a centrifugal pattern since unremodeled (primary) compact bone is present in the innermost regions of some areas (e.g. medial) (Fig 9A and 9H). Secondary osteons are abundant in the inner cortical regions and are especially numerous in the anterolateral, anteromedial and posteromedial areas (Fig 9I). Indeed, in the anterolateral region, they are so extensively developed that they reach the outermost cortex. In the mid and outer cortex, secondary osteons are commonly organized in concentric rows that are closely associated with the LAGs (Fig 9A).

**Fig 10:**
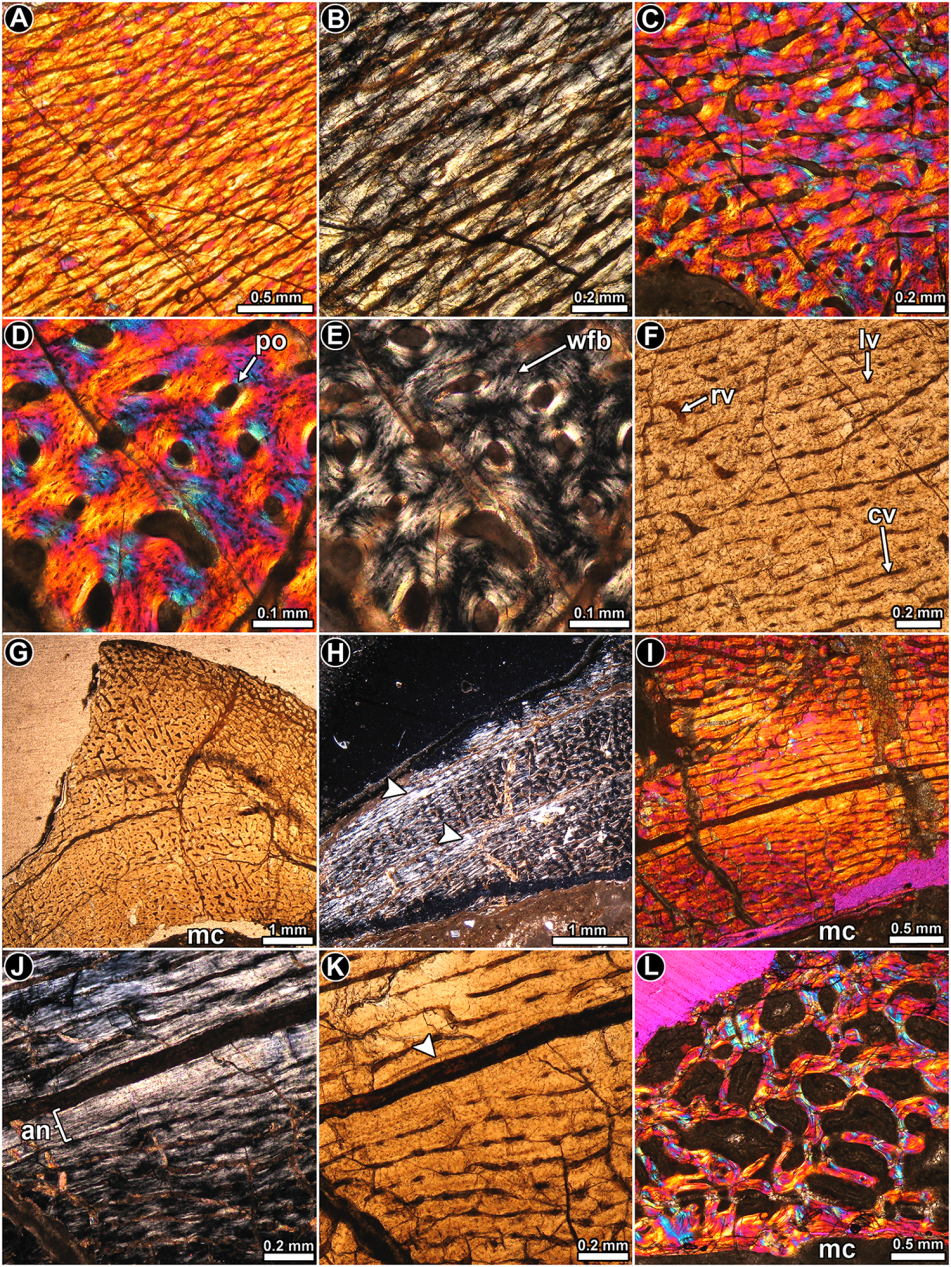
Femur histology of *Leonerasaurus taquetrensis* MPEF 1663. (**A**, **B**) General view (**A**) and detail (**B**) of the primary bone tissue, which is mostly composed of parallel fibered bone. Polarized light with lambda compensator (**A**) and polarized light (**B**). (**C**–**E**) General view (**C**) and detail (**D**, **E**) of coarse woven fibered bone tissue. Polarized (**E**) and polarized light with lambda compensator (**C**, **D**). (**F**) Primary bone tissue in which longitudinally oriented vascular canals predominate. Normal transmitted light. (**G**) General view of the compact bone showing relative abundance of radial vascular canals. Normal transmitted light. (**H**) General view of the cortex in which two poorly defined annuli occur (arrowheads). Note that each annulus exhibits a higher degree of birefringence. Polarized light. (**I**–**K**) General view (**I**) and detail (**J**, **K**) of a single annulus formed in the inner cortex. Note the presence of a single LAG (arrowhead). The broken surface corresponds to a second LAG. Polarized light with lambda compensator (**I**), polarized light (**J**) and normal light (**K**). (**L**) Cancellous bone in the perimedullary region. Bony trabeculae are composed of lamellar bone deposited during different episodes of secondary remodeling. Polarized light with lambda compensator. Abbreviations: an: annulus; cv: circumferentially oriented vascular canal; lv: longitudinally oriented vascular canal; mc: medullary cavity; po: primary osteon; rv: radially oriented vascular canal; wfb: woven fibered bone.

*Leonerasaurus taquetrensis* MPEF 1663: Two partial cross sections where obtained from this specimen: one at the level of the fourth trochanter and the other just above it (these are referred to as the distal and proximal sections from here onwards). Although the outer cortex is eroded away, histological features of the inner and mid cortex can still be discerned. The bone matrix in the proximal section is mostly composed of parallel fibered bone tissue (Fig 10A and 10B), but some variation in this regard occurs. Coarsely bundled woven fibered bone is observed in the inner portion of the posterior and lateral areas (Fig 10C and 10D), as well as in the vicinity of the fourth trochanter in the distal section. Depending on the fibrillar organization in which they are embedded, osteocyte lacunae are rounded or elongated. Vascularization mainly consists of abundant longitudinal and circumferential primary osteons (Fig 10F). Contrary to the observations in other samples, circumferential canals do not form the typical concentric laminae. Radial and oblique canals are also present, but their distribution and density is not uniform. Radial canals are particularly abundant in the distal section at the level of the fourth trochanter, in which they extend from the inner to the outer cortex (Fig 10G).

Regarding the presence of growth marks, at least two annuli are visible in the cortex, with the inner one being more clearly defined than the outer one (Fig 10H). Each annulus consists of poorly vascularized parallel fibered bone, in which their intrinsic fibers exhibit a high degree of organization. At least one LAG is visible in the compact bone (Fig 10I–10K).

Except for the lateral region, the perimedullary cortex of the proximal section exhibits extensive secondary remodeling, and the resorption cavities give the bone a rather cancellous texture. The cancellous spaces are large and they have irregular shapes. The resorption cavities are lined with thin deposits of lamellar bone tissue, which clearly has been deposited during different episodes of remodeling. It is interestingly to note that the innermost cavities are not opened to the medullary cavity (Fig 10L). In some places the medullary cavity is lined by a thin layer of endosteally formed bone.

**Fig 11:**
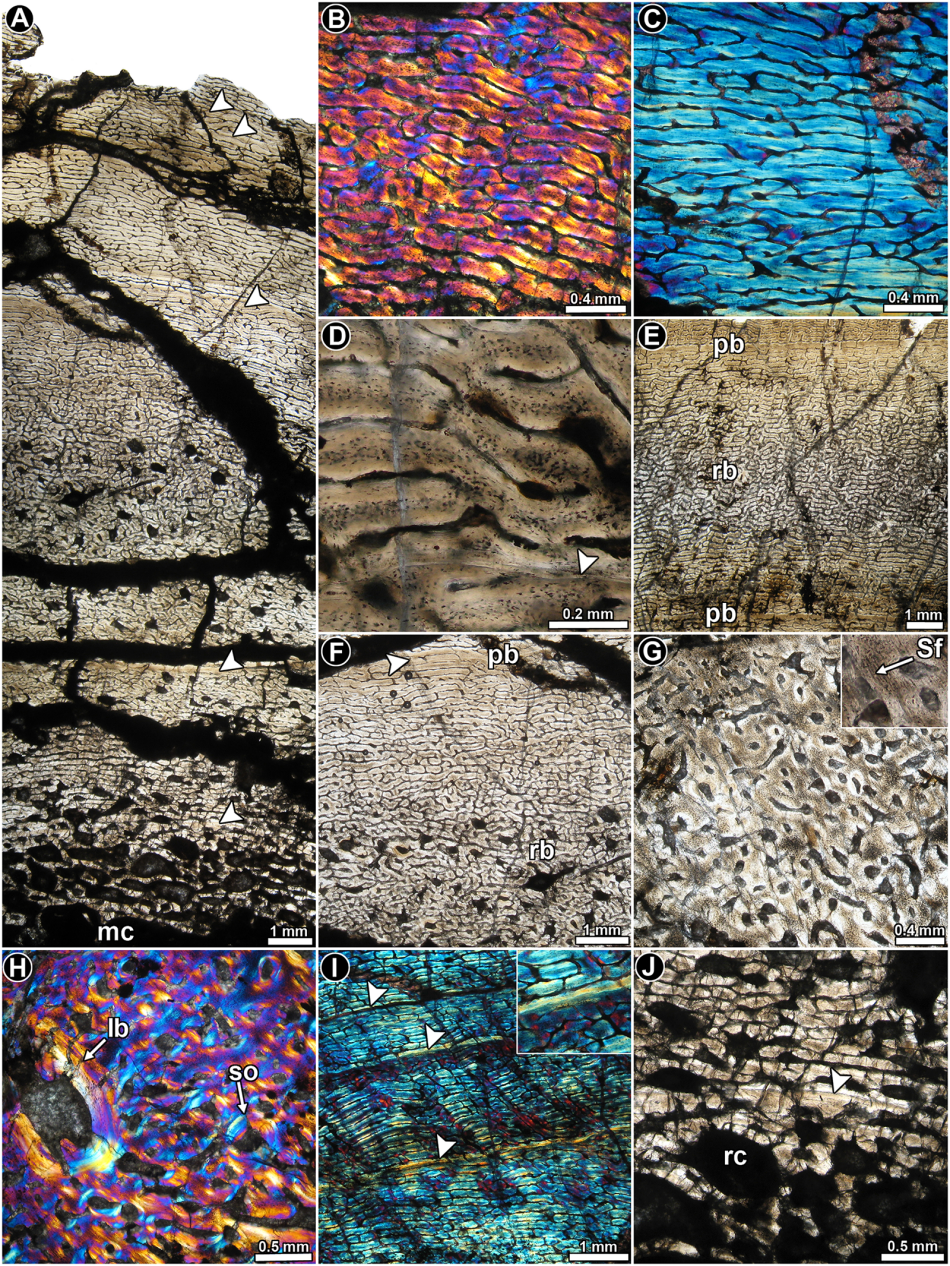
Femur histology of *Lessemsaurus sauropoides* PVL 4822/64. (**A**) General view of the compact bone. Arrowheads indicate LAGs. Normal transmitted light. (**B**) Detail of fibrolamellar bone tissue. Polarized light with lambda compensator. (**C**) Detail of parallel fibered bone tissue. Polarized light with lambda compensator. (**D**) Detailed view of the fibrolamellar complex showing the shape and relative abundance of osteocyte lacunae. Note the presence of a single LAG (arrowhead). Normal transmitted light. (**E**, **F**) General views of the compact bone showing the local variation of the vascularization pattern. Note the transition between plexiform and reticular bone. Normal transmitted light. (**G**) Cortical bone at the anteromedial region of the compacta, in which longitudinally vascular canals predominate. A detailed view of the Sharpey’s fibers is showed in the insert box. Normal transmitted light. (**H**) Detail of the cortical bone at the anteromedial region of the compacta. Note the presence of a large reconstructed erosion cavity lined with lamellar bone. Polarized light with lambda compensator. (**I**) Detail of the primary bone at the mid cortex showing three LAGs (arrowheads). Note the birefringence of the bone tissue accompanying each LAG (box inset). Polarized light with lambda compensator. (**J**) Detailed view of the inner cortex showing a well preserved LAG (arrowhead). Normal transmitted light. Abbreviations: lb: lamellar bone; mc: medullary cavity; pb: plexiform bone; rb: reticular bone; rc: resorption cavity; Sf: Sharpey’s fibers; so: secondary osteon.

*Lessemsaurus sauropoides* PVL 4822/64: This transverse section is composed of a large free medullary cavity enclosed by a thick cortex of compact bone. Although the complete section is badly crushed, histological details are well preserved. In general, the cortical bone is mostly composed of well-vascularized primary bone tissue (Fig 11A). Woven fibered bone tissue dominates the compacta, and with the primary osteons they form the typical fibrolamellar bone tissue (Fig 11B). Parallel fibered bone is also observed, but it is present to a lesser extent than the woven fibered bone (Fig 11C). Osteocyte lacunae in the woven fibered matrix exhibit rounded or slightly flattened shapes and they are in general densely grouped together (Fig 11D). The shape and arrangement of the osteocyte lacunae varies in the parallel fibered bone, where they tend to be more flattened and aligned concentrically.

Vascular canals are profusely distributed in the compact bone and their arrangement departs from the pattern observed in the other dinosaurs studied here. In this regard, vascularization consists of alternating bands of reticular and plexiform bone (Fig 11E and 11F). In the areas dominated by a reticular pattern, the vascular supply consists of a highly convoluted, anastomose-rich network (Fig 11F). On the other hand, the plexiform tissue is characterized by less abundant vascular spaces, with longitudinal and circumferential arrangements and radial anastomoses (Fig 11F). Plexiform bone is more abundant toward the inner cortex. Long radial canals are particularly abundant in the lateral and posterior areas. A conspicuous variation in the vascular canal orientation and density occurs in the anteromedial and lateral areas of the cortex. In these regions the primary vascular spaces are larger but less frequent. They are mostly longitudinally oriented (but never forming laminae), with several oblique anastomoses (Fig 11G). The bone matrix in these regions exhibits a darker appearance, mostly as a result of the relative abundance of both osteocyte lacunae and Sharpey’s fibers (Fig 11G). The extrinsic fibers extend from the inner to the outer cortex. Both anterior and lateral portions of the cortex are also characterized by a relative abundance of secondary osteons, which reach the peripheral regions of the compacta (Fig 11H). All these histological variations are more pronounced at the anteromedial region. Besides the anterior and lateral areas, Sharpey’s fibers are also recorded in other portions of the compacta, but they are never as abundant and densely grouped as in the previously mentioned regions.

Both LAGs and annuli are recorded throughout the compacta (Fig 11A, 11I and 11J). A total of five LAGs (including single and double lines) are counted in the cortex. Each annulus is characterized by poor vascularization or is avascular. They also exhibit a higher degree of arrangement of their intrinsic fibers (i.e. they show strong birefringence) as compared to their neighboring tissues. In general, the zones are thick and they are formed by reticular bone, which changes to a plexiform arrangement before the LAG formation (although in some cases the entire zone is formed by plexiform bone). Since the subperiosteal margin of the cortex is eroded away, we cannot deduce whether an OCL formed in this individual. However, since we do not see a clear reduction in zone thickness toward the outer cortex, we can assume that the individual was actively growing at the time of death.

As previously mentioned, secondary osteons are mostly observed at the anteromedial and lateral areas of the cortex. Several resorption cavities are formed in the inner cortex. Some areas of the perimedullary region exhibit secondary osteons and resorption cavities arranged in poorly defined concentric “laminae”. Unusually large, irregularly shaped resorption cavities are formed in some areas of the inner cortex (e.g. anteromedial)

**Fig 12:**
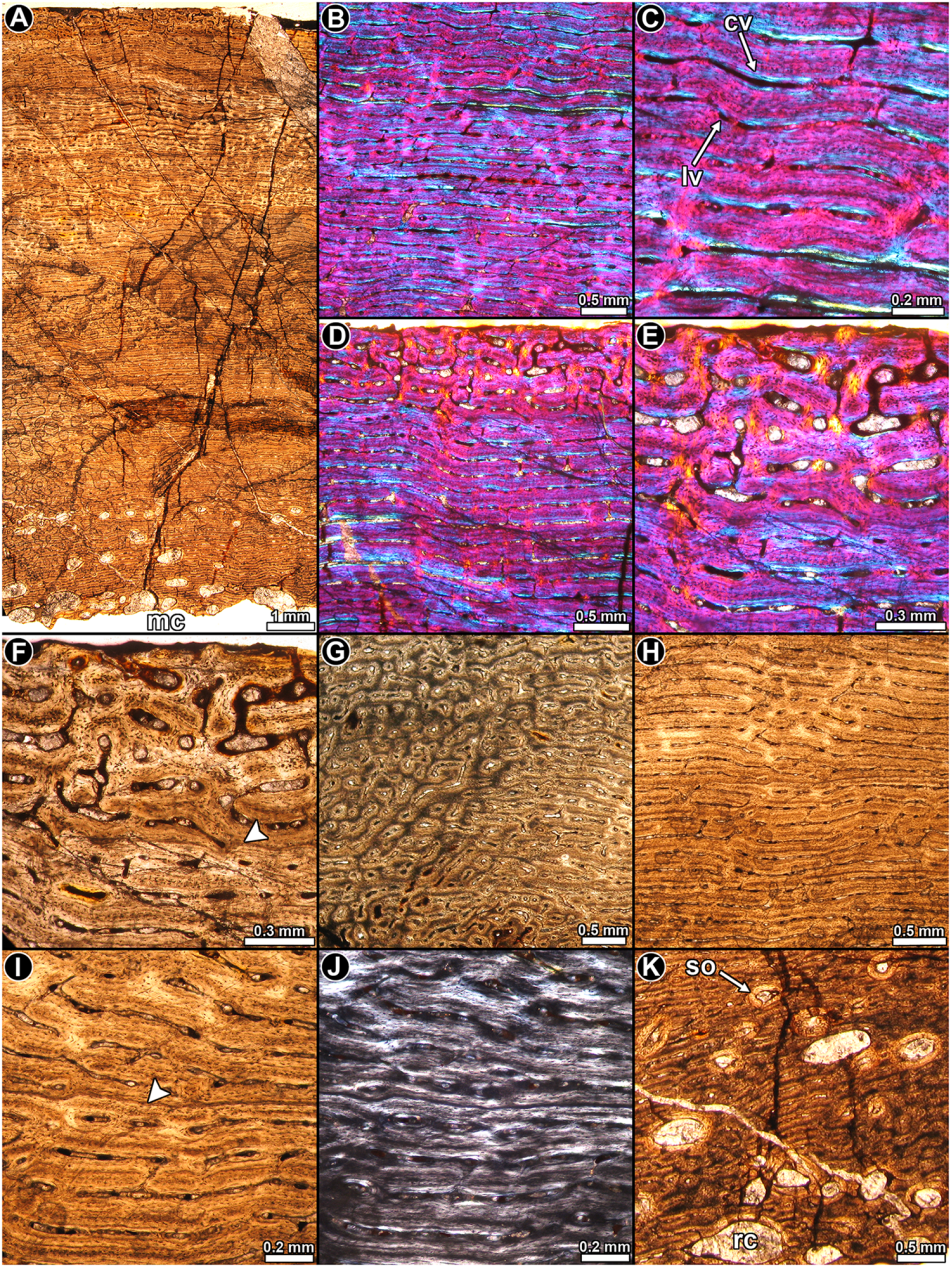
Femur histology of *Volkheimeria chubutensis* PVL 4077. (**A**) General view of the compact bone. Normal transmitted light. (**B**, **C**) General view (**B**) and detail (**C**) of fibrolamellar bone tissue. Polarized light with lambda compensator. (**D**–**F**) General view (**D**) and detail (**E**, **F**) of the outer cortex. Note the striking variation of the vascularization pattern evident in the subperiosteal region. A line of arrested growth is present in the sample (arrowhead). Polarized light with lambda compensator (**D**, **E**) and normal transmitted light (**F**). (**G**) Abrupt transition of the vascularization pattern and bone matrix in the anterolateral portion of the cortex. Normal transmitted light. (**H**–**J**) General view (**H**) and detail (**I**, **J**) of the cortical bone showing a distinct LAG (arrowhead). Normal transmitted light (**H**, **I**) and polarized light (**J**). (**K**) Resorption cavities at the perimedullary region of the cortex. Normal transmitted light. Abbreviations: cv: circumferentially oriented vascular canal; lv: longitudinally oriented vascular canal; mc: medullary cavity; rc: resorption cavity; so: secondary osteon.

*Volkheimeria chubutensis* PVL 4077: The cortical bone is mainly formed by highly vascularized, fibrolamellar tissue (Fig 12A and 12B). In some areas, the intrinsic fibers become slightly organized, but they do not form a well-defined, parallel fibered, matrix. The osteocyte lacunae exhibit a rather globular and disorganized arrangement in the woven matrix (Fig 12C). Vascular canals are mostly longitudinally and circumferentially oriented in concentric laminae (Fig 12A and 12B). Radial anastomoses are regularly observed in the cortex. These canals are short, and link osteons of no more than two successive laminae. Slight changes in the vascularization pattern (i.e. variation in the abundance of longitudinal and circumferential canals) produce an ill-defined stratification in the cortex, which is best recognized under low magnifications. At least eight of these “growth” cycles (i.e., modulations) are present in the compacta. A striking change in the vascularization pattern occurs in the subperiosteal surface, in which the well organized pattern of concentric laminae is lost and the vascular spaces exhibits a plexiform pattern (Fig 12D–12F). The osteocyte lacunae are also more densely grouped in this layer, in which the intrinsic fibers are more disorganized than in the bone formed just below it. Another important change in the vascularization pattern is observed from the inner toward the outer cortex in the anterolateral region of the compacta (Fig 12G). As in the thin layer of subperiosteal cortex described above, in this area the concentric arrangement of vascular spaces is lost. Primary osteons are mostly longitudinally and obliquely arranged. The osseous matrix in this portion of the section shows a high density of osteocyte lacunae and Sharpey’s fibers. These extrinsic fibers form at an oblique angle to the outer surface. Sharpey’s fibers are also present in the inner portion of the lateral cortex, but they are much less abundant.

Growth marks are uncommon and they are mostly recognized in the outer mid compacta (Fig 12H–12J). Erosion cavities form around the mid cortex and they tend to form in rows. Some erosion cavities are lined with lamellar bone; but only a few secondary osteons occur (Fig 12K).

**Fig 13:**
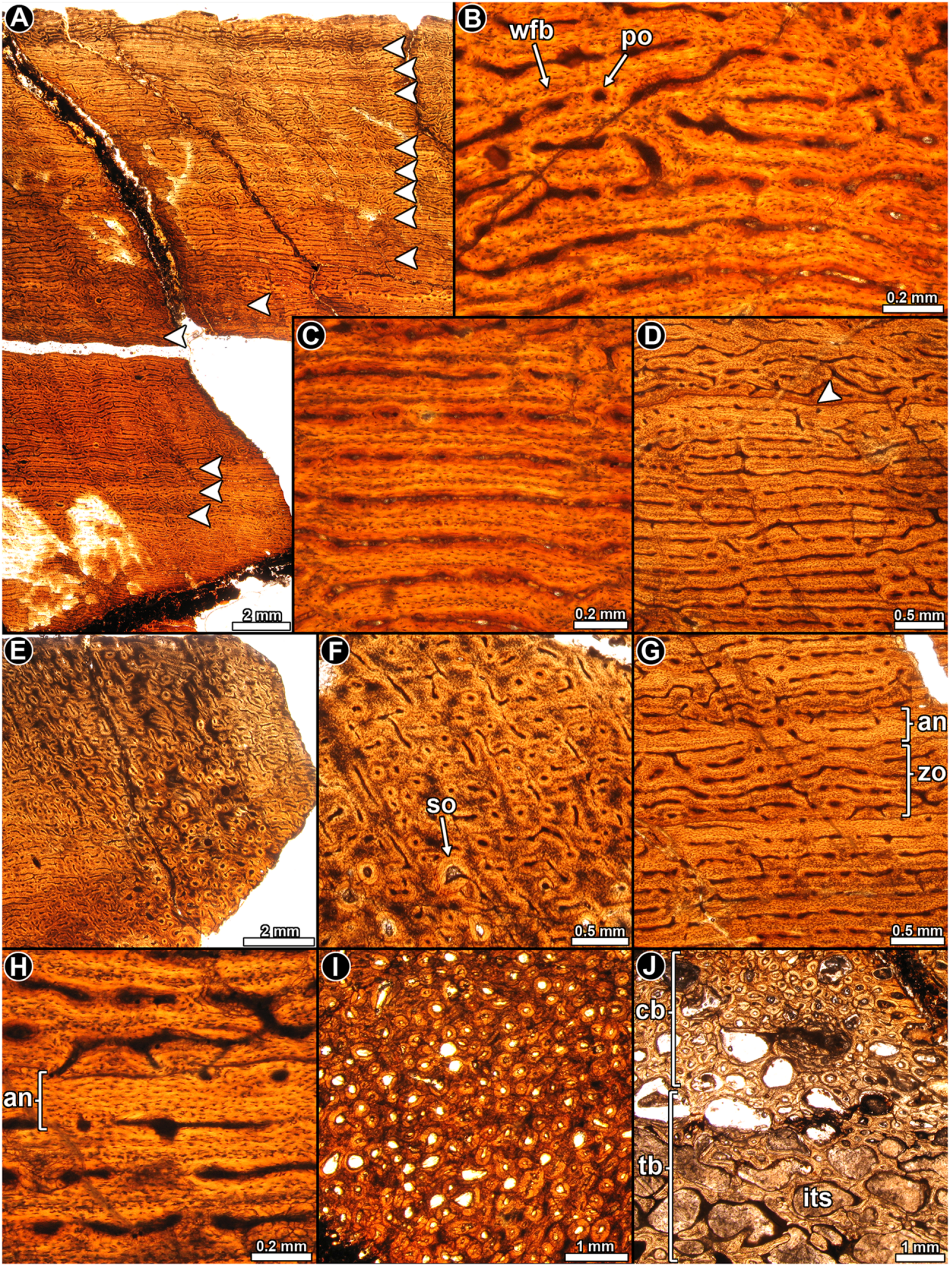
Femur histology of *Patagosaurus fariasi* PVL 4075. (**A**) General view of the compact bone. Note the stratified pattern of the primary bone. Normal transmitted light. (**B**, **C**) Details of the fibrolamellar complex. Note the high density of osteocyte lacunae. Normal transmitted light. (**D**) Detail of laminar bone tissue. The primary bone is interrupted by a single LAG. Normal transmitted light. (**E**, **F**) General view (**E**) and detail (**F**) of the cortical bone at the anterolateral region of the bone. Note the abrupt variation in the vascularization pattern and the relative abundance of secondary osteons. Normal transmitted light. (**G**, **H**) General view (**G**) and detail (**H**) of the annuli in the cortical bone. Note the variation in the density, shape and arrangement of the osteocyte lacunae. Normal transmitted light. (**I**) Haversian bone tissue at the anterolateral portion of the compacta. Normal transmitted light. (**J**) Transition between the trabecular and compact bone in the perimedullary region. Normal transmitted light. Abbreviations: cb: compact bone: an: annulus; its: intertrabecular space; po: primary osteons; so: secondary osteons; tb: trabecular; wfb: woven fibered bone; zo: zone.

*Patagosaurus fariasi PVL 4075*: Highly vascularized fibrolamellar bone tissue is predominant in the cortex (Fig 13A), and the intrinsic fibers of the woven matrix becomes slightly organized toward the outer cortex. Osteocyte lacunae are densely distributed in the matrix (Fig 13B). Vascularization predominantly consists of concentric rows (laminae) of longitudinal and circumferentially arranged primary osteons, which are anastomosed by radial and oblique canals (Fig 13B–13D). Such vascular pattern, however, is not homogeneous in the entire cortex, and several local variations occur. For example, the density of radial and oblique canals varies in different areas, and patches of reticular bone are observed in some areas of the cortex. A distinctive variation occurs at the anterolateral portion of the compacta, in which large longitudinal and oblique canals predominate (Fig 13E and 13F). These canals do not form in typical laminae and they appear to be more distantly spaced than in the other areas of the compacta. The oblique and longitudinally arranged osteons seem to be larger than those located in the other portions of the compacta and they occupy an area that extends from the anterolateral region of the outer cortex toward the lateral side of the inner cortex. The bone matrix in this area contains a high density of osteocyte lacunae, which give a darker color to the tissue (Fig 13F).

A clear pattern of stratification in the cortex is observed under low magnifications, which consist of dark “zones” and lighter “annuli” (Fig 13G). The lighter appearance of these “annuli” corresponds to the low density of the osteocyte lacunae, which in some cases exhibits some spatial arrangement (Fig 13H). A slight degree of organization is present in the intrinsic fibers of some annuli, but they do not exhibit the typical mass birefringence of the parallel fibered bone. A minimum of fourteen “annuli” are formed in the compacta (Fig 13A). A single LAG occurs in the outermost cortex, and it is followed by a narrow region of vascularized bone in a slightly more organized matrix (Fig 13D). No OCL is evident in the cortex.

Secondary remodeling is evident in the perimedullary cortex. Several resorption cavities and secondary osteons are formed in the anterolateral portion of the sample. In this area, many generations of secondary osteons extend to the mid cortex and some of them even reach the outer cortex (Fig 13I). Their distribution coincides with the particular primary bone describe above. A small portion of cancellous bone is preserved in the sample. Bony trabeculae are formed by secondary lamellar bone deposited during different episodes of remodeling (Fig 13J).

## Discussion

### Bone histology and ontogenetic stages

Specimens PVL 3526 and PVL 3669 of *Riojasaurus* are smaller than the largest known individual (PVL 3808), and their femoral lengths are respectively 79% and 95% of the femur length of PVL 3808. However, it is worth noting that evidence of somatic maturity (an OCL) is present in specimen PVL 3526. A clear reduction in the spacing between successive growth marks is not evident in specimen PVL 3669, suggesting that sexual maturity had not yet been attained in this individual. Unfortunately, the lack of axial parts of the skeleton for this individual precluded us from assessing the degree of neurocentral suture closure.

*Coloradisaurus* PVL 5904 is more than twice as large as the holotype (PVL 3967). Although this specimen exhibits complete neurocentral suture closure in all the preserved vertebrae (one cervical, all the dorsals, and three caudals), the absence of a distinct OCL suggests that somatic maturity was not reached. Distinct changes in intrinsic fiber orientation and growth mark spacing are not evident in the cortex. We must note, however, that the poor preservation of the tissue could mask some important histological changes related to the intrinsic fiber orientation and distribution of the growth marks.

The sampled individual of *Adeopapposaurus* is about 77% the size of the largest known specimen, and accordingly the neurocentral sutures of the sacral and anterior caudal vertebra are fully open, and the transverse processes and sacral ribs are also unfused. The absence of an OCL is congruent with these anatomical features, although a distinct increase in the intrinsic fiber arrangement in the outer cortex (Fig 5C) suggests that sexual maturity may have already been attained.

The *Massospondylus* specimen BP/1/4934 represents the largest of all known *Massospondylus* specimens [47] and is larger than all the individuals previously histologically sampled by Chinsamy [8]. Despite this large size, a distinct OCL is not present in the outer cortex, suggesting that this individual had not yet reached somatic maturity. However, both the reduction in the spacing between growth marks and the increase in the intrinsic fiber organization suggest that the individual was sexually mature at the time of death. It is interesting to note that the posterior dorsal vertebrae exhibit a partially closed neurocentral suture. The histological features recorded in *Massospondylus* are identical to those observed in *Leyesaurus* PVSJ 1079, which indicate similar ontogenetic stages (i.e. sexually mature but not yet somatically mature) [39].

In both individuals of *Mussaurus* the subperiosteal bone has been partially eroded and we cannot be certain whether an OCL had formed. Irrespectively, both individuals possess relatively closely spaced growth lines in the outer third of the cortex, which is even more pronounced in specimen MPM-PV 1815. Despite the large size of *Mussaurus* MLP 61-III-20-22 (the largest known specimen for this taxon), the neurocentral sutures of the dorsal vertebrae are only partially closed, as occur in *Massospondylus* BP/1/4934. Although the size of the specimen MPM-PV 1815 is approximately 69% of MLP 61-III-20-22 (based on femur circumference), both specimens appear to be at a similar ontogenetic stage (i.e. sexually mature though not yet somatically mature).

In the *Leonerasaurus* holotype most of the outer cortex has been eroded, which impedes the deduction of whether an OCL was present or not. Nevertheless, according to Pol et al. [22], the complete closure of the neurocentral sutures in most presacral and sacral vertebrae indicate that the specimen was nearly fully-grown.

The outermost cortex of the sampled *Lessemsaurus* is not preserved, which precludes the identification of an OCL. However, the absence of a reduction of the spacing between preserved growth marks in the outer cortex suggests that the individual was still actively growing at the time of its death. The fully opened condition of the neurocentral sutures of the cervical and dorsal vertebrae supports this inference. The distinctive variation of the bone histology of *Lessemsaurus* from other sauropod dinosaurs (e.g. Neosauropoda) precludes the usage of the HOS proposed by Klein and Sander [34] to deduce sexual maturity.

In the case of *Volkheimeria*, this specimen lacks an OCL and closely spaced growth marks, which concurs with its relatively small size and, more importantly, with the completely fully open neurocentral sutures. The usage of HOS defined by Klein and Sander [34] is not straightforward since the histological types that define each HOS are mostly based on qualitative and relative features (e.g. the differences between tissues type E and D is the larger size of vascular canals in the former). Nevertheless, according to the cortical tissue type, *Volkheimeria* appears to correspond to type E, which is characteristic of Klein and Sander’s [34] HOS 9 and 10, which implies that the individual was at a sexually mature ontogenetic stage.

The cortex of the sampled *Patagosaurus* does not have an OCL, but exhibits regularly spaced “annuli”, which are more pronounced in the outer cortex. As shown in Fig 13A, there is no clear reduction in the spacing between growth marks towards the periphery. The cortex of *Patagosaurus* exhibits features of Klein and Sander’s [34] HOS 10 and 11, for which sexual maturity has been reached. Therefore, the ontogenetic stage of the sampled *Patagosaurus* appears to be at a more advanced stage than that recorded in *Volkheimeria*.

Thus, our histological results support the gross anatomical findings that our study sample is representative of sexually mature individuals that had not yet reached somatic maturity. The only exception to this is *Riojasaurus* PVL 3526, which appears to have attained somatic maturity. Interestingly, we found some incongruence in terms of the inferred ontogenetic stage and the relative size of some taxa. For example, in both *Riojasaurus* and *Mussaurus* we found that the more advanced ontogenetic stages are recorded in the smaller specimens for each taxon. This variation could be due to several different causes, including sexual dimorphism, presence of cryptic species or, as proposed for the basal sauropodomorph *Plateosaurus*, developmental plasticity [16; 39].

Another important result that arose from the comparative data is related to the timing of the attainment of sexual and somatic maturity and the closure of the neurocentral sutures. Our data shows that neurocentral suture is open in different parts of the vertebral column in sexually mature individuals of both basal sauropodomorphs and the more derived sauropods taxa (e.g. *Adeopapposaurus* and *Volkheimeria*). The presence of scars of the neurocentral suture of large bodied (but somatically immature) individuals (e.g. sampled specimens of *Mussaurus* and *Massospondylus*) indicates that the complete closure of neurocentral sutures in the vertebral column was achieved late during ontogeny, and well after the attainment of sexual maturity. This pattern agrees with that reported in extant pseudosuchian archosaurs [38, 48], and with Carballido & Sander’s [49] proposal that sexual maturity in sauropods is attained before the complete closure of the neurocentral sutures. General interpretations pertaining to ontogenetic growth should be supported by a large sample of representatives of different ontogenetic stages of each species. Despite this limitation our findings provide insight into the relationship between bone histology and the attainment of sexual and skeletal maturity of sauropodomorph dinosaurs [39, 49].

### Variations in the growth dynamics of basal sauropodomorpha and Sauropoda

Histological features of the primary bone, such as the organization of the intrinsic fibers, presence and distribution of growth marks and density and arrangement of vascular spaces, provide much information about the rate of periosteal bone accretion, and hence the growth rate of vertebrates [29, 30, 50]. In this sense, a densely vascularized bone matrix with highly disorganized intrinsic fibers (woven fibered bone) grows relatively faster than poorly vascularized matrix formed by well-organized intrinsic fibers (parallel fibered and lamellar bone) [29, 30, 50]. The presence of regular growth marks in the cortical bone is interpreted as periodic interruptions (i.e. lines of arrested growth) and/or a decrease in the rate of bone apposition (i.e. annuli) that result from endogenous cycles of growth reinforced by natural environmental cycles [51]. The evaluation of these parameters permits a characterization and comparison of the growth dynamics of the sampled sauropodomorph taxa.

**Fig 14:**
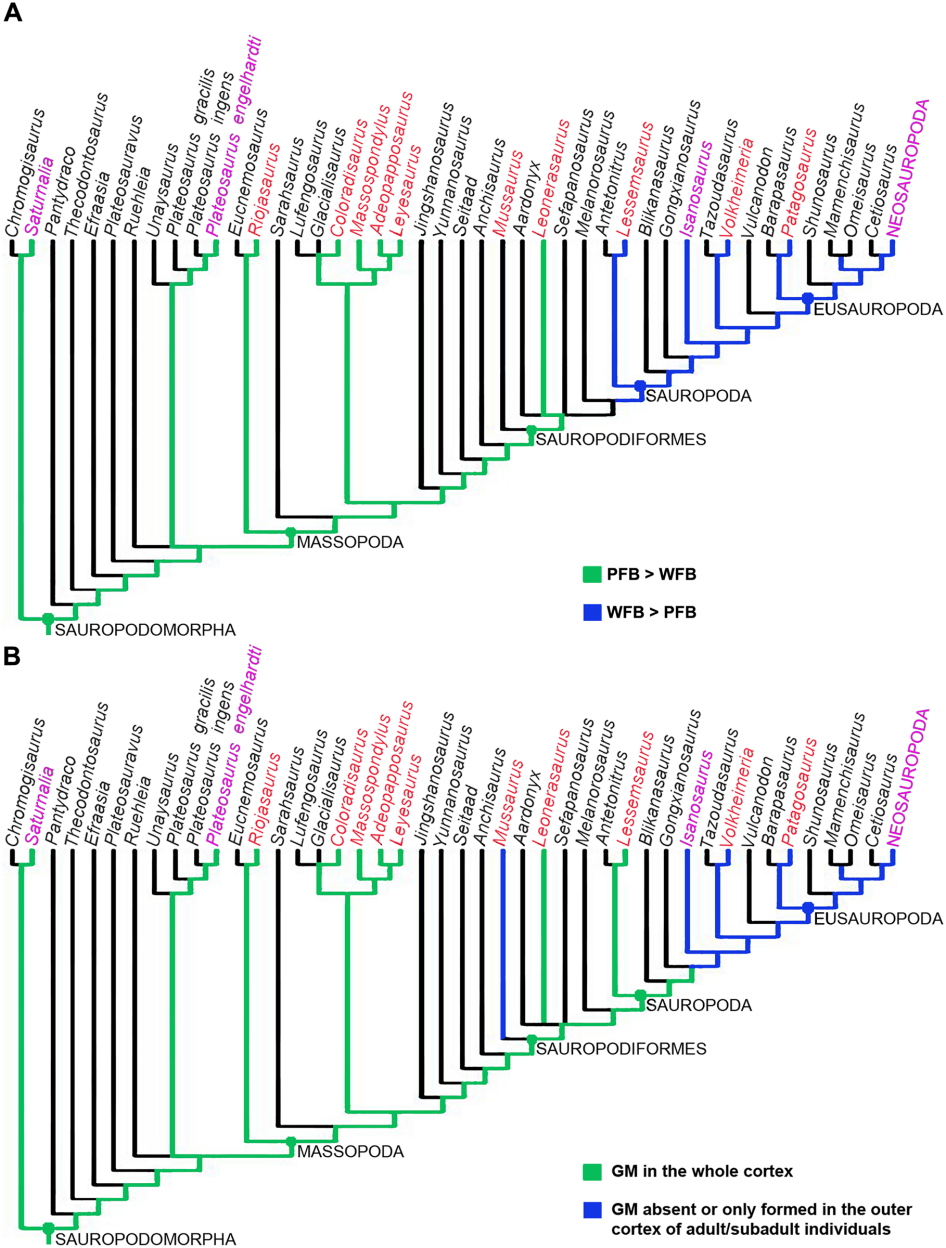
Optimization of the histological characters in the phylogeny of basal sauropodomorpha. (**A**) Optimization of the character “predominant bone tissue in the compact bone”. This character refers to the relative proportions of woven fibered (WFB) and parallel fibered bone (PFB). (**B**) Optimization of the character “growth marks (GM) in the femoral cortex”. The characters have been optimized for the reduced strict consensus tree from the modified matrix of Otero et al. [40]. Except for those taxa studied here (red lettering), the data for the histological scoring (magenta lettering) were obtained from the following sources: *Saturnalia* [17, M. Sander com. pers.], *Plateosaurus engelhardti* [16, M. Sander com. pers.], *Isanosaurus* [3], and Neosauropoda [2, 34]. Distinction between stem and node based taxa were not indicated.

The predominance of parallel fibered bone tissue in the whole cortex of most of the sampled basal sauropodomorphs implies a relatively slower growth rate in these taxa as compared to most sauropods, which have an abundance of woven fibered bone tissue in their cortices. Although woven fibered bone was formed in the femoral cortices of basal sauropodomorphs sampled here, this tissue was commonly restricted to the inner cortex, and therefore representative of the earlier stages of growth. Using the relative proportion of woven fibered and parallel fibered bone in the cortex as a proxy for the relative growth rate in basal sauropodomorphs, the evolution of this parameter can be assessed. As shown in Fig 14A, the optimization of the character suggests that slow growth rates (i.e. predominance of parallel fibered bone) is plesiomorphic for Sauropodomorpha, while the derived condition of faster bone depositional rates is a synapomorphy of Sauropoda, which agrees with the hypothesis as outlined in [3]. The ambiguity reported in *Mussaurus* is related to the polymorphic condition of this character (i.e. in MPM-PV 1815, parallel fibered bone predominates over woven fibered bone, while in MLP 61-III-20-22 the inverse condition is observed). Although, as previously mentioned, an osseous pathology has been reported in *Mussaurus* MLP 61-III-20-22, this condition is clearly temporally unrelated to the high amount of woven fibered bone tissue observed in most of the cortical bone. The unusual bone tissue described in this specimen is endosteally formed and it is located within the medullary cavity and in the perimedullary region [46], which suggests that it occurred late during ontogeny. This contrasts with the occurrence of the periosteally formed woven fibered bone tissue in the perimedullary region, which indicates that this primary tissue began to be deposited from early stages of ontogeny onwards. Thus, we are confident that the high amount of woven fibered bone that predominates in *Mussaurus* MLP 61-III-20-22 is unrelated to the pathology, and indicates that the capability to maintain high, sustained growth rates developed before the origin of Sauropoda.

It is evident that all basal sauropodomorphs sampled in previous studies [3, 8, 16] have growth marks throughout the entire cortex indicating cyclical growth dynamics (Fig 14B). Our study revealed that *Mussaurus patagonicus* is an exception to this general pattern among basal sauropodomorphs. Although both sampled specimens exhibit growth marks, these are restricted to the outer third of the cortex. This histological organization suggests that growth in *Mussaurus* only became cyclical during the later phases of its ontogeny, possibly after sexual maturity was reached.

As previously mentioned, another important feature of *Mussaurus* is recorded in specimen MLP 61-III-20-22, which exhibits higher amounts of woven fibered bone as compared to all other basal sauropodomorphs studied, and even as compared to the other sampled specimen of *Mussaurus* (MPM PV 1815). This histological pattern reveals a high and sustained growth rate during most of the development of *Mussaurus* MLP 61-III-20-22. The extended rapid phase of growth recorded in this specimen possibly allows for the rapid attainment of its large body size. Interestingly, specimen MPM PV 1815 has a shaft circumference of 22.2 cm as compared to 32 cm in MLP 61-III-20-22. The differences observed in the amount of woven fibered bone in the compacta of the two sampled specimens of *Mussaurus* reveals intraspecific variation, which could be attributable to sexual dimorphism and/or developmental plasticity [7, 16]. If developmental plasticity occurs in *Mussaurus*, such plasticity could be related to the attainment of high and sustained growth rates in some individuals of this taxon. As inferred for *Plateosaurus* [7, 16], developmental plasticity can only be supported if a continuous variation in terms of adult size and growth rate is evident in a larger sample of *Mussaurus*.

In the case of sauropod dinosaurs (with the exception of *Lessemsaurus*), the predominance of uninterrupted and highly vascularized fibrolamellar bone in *Volkheimeria* and *Patagosaurus* is indicative of rapid, sustained growth rates, which agrees with previous studies of sauropods (e.g. [3, 15, 34, 52]). Although some histological differences are detected between these taxa, such variation appears to be related to ontogeny. The cyclical growth pattern observed in *Lessemsaurus*, contrasts with that of other sauropod dinosaurs. In *Lessemsaurus*, periodic lines of arrested growth and annuli interrupt the deposition of highly vascularized fibrolamellar bone with reticular vascular organization, suggesting that although cyclical growth occurred, growth was accelerated during the fast growing periods. Our findings show that this basal sauropod retains the plesiomorphic pattern of cyclical growth, but during the periods of rapid growth (even at sub-adult stages) it grew at rates equivalent to that of the derived sauropods. This is perhaps not unexpected since *Lessemsaurus* is considered to be a transitional form in the origin of Sauropoda, showing both derived and plesiomorphic anatomical features [19]. It is highly improbable that the presence of growth cycles are related to body size since small bodied taxa (e.g *Adeoppaposaurus*, femur circumference, fc, equals 7.6 cm) exhibit growth marks in the whole cortex as do larger body sized taxa (e.g. *Lessemsaurus*, fc: 36 cm). In the same way, growth marks restricted to the outer cortex are found in specimens having a wide range of sizes (*Patagosaurus*, fc: 61 cm and *Mussaurus* MPM PV 1815, fc: 22.2 cm).

### Early evolution of sauropodomorph growth dynamics

Sander et al. [3] proposed that the origin of Sauropoda was accompanied by a significant change in their growth strategy that facilitated the attainment of gigantism. Based on the then available data, they postulated that non-sauropod sauropodomorphs (i.e., *Plateosaurus* and *Massospondylus*) were characterized by having a cyclical growth pattern, whilst the more derived sauropods (Eusauropoda) and an individual of the basal Triassic sauropod cf. *Isanosaurus* experienced sustained rapid growth during most of their ontogeny [2, 3]. These authors concluded that sustained and rapid growth was important for the attainment of gigantism in the clade [2, 3]. Our comprehensive sampling that includes seven additional basal sauropodomorphs and three sauropods, as well as the previously published taxa (see Table 4), reveals a higher variation with regard to the growth dynamics among sauropodomorph dinosaurs, particularly among the basal (non eusauropods) taxa.

**Table 4:** Growth mark record in femora (Fem) and humeri (Hum) of sauropod dinosaurs.

Taxon	Element*	LAGs and/or annuli	Reference
*Giraffatitan*	Hum, fem	Present (outer cortex)	[15]
*“Barosaurus”*	Hum, fem	Absent (“type A”) or present throughout cortex (“type B”)	[15]
*Dicraeosaurus*		Absent	[15]
Indet. mamenchisaurid	Hum	Present (outer cortex)	[80]
*Janenschia*	Hum, fem	Present (outer cortex)	[15]
*Apatosaurus*	fem	Present (outer cortex)	[80]
*Camarasaurus*	Hum, fem,	Present (outer cortex)	[34, 80]
*Bothriospondylus*	Hum, fem	Present, but unclear distribution	[14, 54]
*Europasaurus*	Fem	Present throughout cortex	[13]
*Ampelosaurus*	Hum, fem	Present, but unclear distribution**	[56]
*Phuwiangosaurus*	Fem, hum,	Present, but unclear distribution**	[55]
*Magyarosaurus*	Fem, hum	Present, but unclear distribution**	[73]
*Lirainosaurus*	Fem	Present, but unclear distribution**	[59]
*Alamosaurus*	Fem, hum	Present (outer cortex) or absent*	[56, 57]
*Dreadnoughtus*	Hum	Present, but unclear distribution**	[81]
*Paluxysaurus*	Hum, fem	Present, but unclear distribution**	[75]
*Chubutisaurus*	Fem	Present, but unclear distribution**	[58]
*Bonitasaura*	Fem, hum,	Present (outer cortex)	[74]
cf. *Isanosaurus*	Hum	Absent*	[3]

*only subadult sampled

** refers to specimens without histological detail; or ones that have extensive secondary reconstruction and the original primary bone cannot be determined.

Our results show that the basal sauropodomorph *Mussaurus* has a growth pattern previously ascribed to sauropods i.e. growth rings occur only in the outer cortex [2, 3]. Furthermore, the putative basal sauropod, *Lessemsaurus* possesses the ‘typical’ “prosauropod” growth strategy, with growth marks evident throughout the cortex. Additionally, our analysis and data from the published literature, shows the existence of at least two distinctive growth patterns in the clade:

1. Cyclical growth during most of ontogeny as indicated by growth marks occurring throughout the cortex.
2. Uninterrupted, sustained growth during the early ontogenetic stages (absence of growth marks), but the onset of cyclical growth during later ontogeny (i.e. growth marks in the outer cortex of adult individuals).

The first growth pattern occurs in several basal sauropodomorphs [7; 8; current study], as well as in the basal sauropod *Lessemsaurus* and the dwarf sauropod *Europasaurus* [13]. The second growth pattern (the ‘typical’ Sauropod growth strategy) is predominantly found amongst Sauropoda [2, 3], (Table 4), but also characterizes the ontogenetic growth of the basal sauropodomorph *Mussaurus*. It should be noted, that the diplodocid *Dicraeosaurus hansemani* is the only sauropodomorph that has a growth pattern of sustained uninterrupted growth [15], which could represent a third growth pattern among the Sauropodomorpha. However, M. Sander (personal communication, 2017) suggests that this histological description may be unreliable because of the small sample size, and limited size range of *Dicraeosaurus* studied.

Our data agrees with Sander et al. [2, 3] that during the evolution of Sauropodomorpha, a distinctive change in the growth dynamics (from cyclical to uninterrupted growth) occurred. The question however, remains as to the onset of the ‘typical’ sauropod growth pattern in the evolutionary history of Sauropodomorpha. Our finding of this growth strategy in the Early Jurassic basal sauropodomorph *Mussaurus* and the occurrence of this tissue in the Triassic early sauropod *Isanosaurus* [3] could be interpreted in two ways: (i) the potential for such a growth strategy could have arisen among Sauropodomorpha before the origin of Sauropoda in the Triassic (*i*.*e*. synapomorphic trait of the more inclusive clade Sauropodiformes (sensu [53]) for both the Sauropoda and the derived basal sauropodomorphs, (ii) the ‘typical’ sauropod growth strategy could have arisen independently in the basal sauropodomorph *Mussaurus*, and in the sauropods (i.e., a homoplastic trait). The optimization of the character based on the growth strategy in sauropodomorph dinosaurs (Fig 14B) indicates that the second scenario is more parsimonious. This suggests that the ‘typical’ sauropod growth dynamics appeared at least twice in the evolution of the Sauropodomorpha. Furthermore, our comprehensive examination of the bone histology and growth dynamics of several sauropodomorph dinosaurs, also revealed that the large-bodied sauropod *Lessemsaurus* lacks the ‘typical’ sauropod growth pattern [2, 3]. Thus, it appears that the ‘typical’ sauropod growth pattern is not an autapomorphic condition for Sauropoda.

### Distribution of cancellous bone and Sharpey’s fibers

Besides those histological features directly related to the growth dynamics of sauropodomorph dinosaurs, we found some microstructural characteristics that deserve further discussion. Interestingly, whereas in all non-sauropod sauropodomorph dinosaurs and the basal sauropod *Lessemsaurus* the femoral medullary cavity is free, this cavity is filled with cancellous bone in the two eusauropods analyzed here, *Volkheimeria* and *Patagosaurus*. This distribution pattern of cancellous bone in sauropodomorph dinosaurs is consistent with other published data [3, 15, 16, 52, 54–61]. This observation has two main implications. Firstly, although the presence of cancellous bone within the medullary cavity has been considered to lack a strong phylogenetic signal in tetrapods [61], this does not appear to be true for sauropodomorph dinosaurs. Secondly, the absence of cancellous bone in the medullary cavity of long bones shafts is optimized as the plesiomorphic condition in Sauropodomorpha. The derived condition (i.e., medullary cavity filled with cancellous bone) does not represent a synapomorphy of Sauropoda but given its absence in *Lessemsaurus* it most likely appeared closer to Eusauropoda. As pointed out by previous authors, the cancellous structure of the medullary cavity could be biomechanically advantageous (e.g., absorbing impact energy during locomotion) and/or for providing scaffolding to support the bone marrow [61, 62]. The adaptive benefits of cancellous bone may therefore explain the acquisition of this character in large bodied sauropods, and this was perhaps later retained in smaller bodied forms (e.g., the titanosaur *Lirainosaurus atibiae* [59]) through phylogenetic inertia.

A second histological feature worth mentioning is related to the distribution of Sharpey’s fibers in the analyzed samples. These extrinsic fibers are more densely grouped in the anterior portion of the cortices of *Riojasaurus*, *Coloradisaurus*, *Mussaurus*, *Lessemsaurus*, *Volkheimeria*, and *Patagosaurus*, in which they obscure the nature of the intrinsic fibers and extend from the inner to the outer regions of the cortex. The extensive amount of Sharpey’s fibers is associated with a strong modification in the vascular canals density and organization. In *Mussaurus*, *Lessemsaurus*, and *Patagosaurus*, this “radial patch” of modified primary bone is also accompanied by a larger degree of secondary remodeling. A patch of modified primary bone with extensive remodeling is also observed in the cortex of *Massospondylus*, but its relative position cannot be assessed with certainty. The modified area of the cortex is not located exactly in the same place in all the samples. In *Riojasaurus*, and *Coloradisaurus* it is restricted to the anterior quadrant of the compacta. In *Mussaurus* it is clearly observed in the anteromedial and anterolateral regions, being more pronounced in the latter. Finally, in *Lessemsaurus*, it is located on the anteromedial and lateral regions, whilst in *Volkheimeria* and *Patagosaurus* it is restricted to the anterolateral area.

According to previous interpretations [46], the large amount of Sharpey’s fibers is possibly related to the insertion of the femorotibialis muscles [63–69]. Furthermore, besides the presence of a higher amount of Sharpey’s fibers, the abrupt change in the vascularization pattern also suggests an area of muscle attachment [69]. The presence of at least two patches of modified primary bone with associated remodeling in *Mussaurus* and *Lessemsaurus* is therefore most likely related to the insertion of the internal and external components of the femorotibialis muscle. The presence of both femorotibialis muscles has been previously inferred in the basal sauropodomorphs *Massospondylus*, *Plateosaurus* and *Saturnalia* [63, 65, 68]. The absence of a distinct region of modified primary bone in *Adeopapposaurus*, *Leyesaurus* and *Leonerasaurus* is possibly related to the location of the thin section along the shaft, rather than a true absence. As mentioned in the Materials and Methods section, the fragmentary nature of the *Leonerasaurus* sample made it impossible to obtain a thin section at the midshaft level (and instead, our sections were obtained at the level of the fourth trochanter and above). In the case of *Leyesaurus* and *Adeopapposaurus*, although the samples where obtained from the midshaft, it must be noted that this region is not entirely homologous in all basal sauropodomorphs, since there appears to be considerable variation in the relative position of the fourth trochanter [70]. In other words, whereas the midpoint of the *Leyesaurus* femur (i.e. the midshaft) is located far distally from the fourth trochanter, the same point is positioned just below the fourth trochanter in *Mussaurus*. Whereas the samples of *Adeopapposaurs* and *Leyesaurus* were obtained distally from the fourth trochanter, all the other specimens (with the exception of *Leonerasaurus*) were sampled just below this protuberance. We predict that a sample obtained in the homologous region in *Adeopapposaurus*, *Leyesaurus*, and *Leonerasaurus* (and other basal sauropodomorphs) will exhibit the same histological feature.

The femorotibialis muscles commonly occupy most of the femoral shaft and possess a direct insertion in the bones, i.e. the muscle fibers contact the bone without tendinous mediation [69]. This type of muscular insertion commonly leaves no macroscopic traces (e.g. scars or rugosities) on the bone surface [69]. It is interesting to note that, despite the broad area occupied by the mm. femorotibiales in the archosaurian femora [69], the only strong histological evidence for this soft tissue is observed in a restricted portion of the cortex. This kind of variation has been documented in extant vertebrates, including archosaurs, where a muscle leaves histological traces (i.e. Sharpey’s fibers) only in the part where it is inserted [69]. Such a pattern has been explained as a result of the strength of the tensile forces of a muscle acting differently on different parts of a bone. In this sense, a large amount of Sharpey’s fibers are recruited in those regions where the muscle imparts high forces on the bone [69]. Accordingly, the modified primary bone in the Sauropodomorpha is linked to the portions of the bone wall subjected to high tensile forces from these muscles. This hypothesis is further supported by the high incidence of secondary osteons in these areas, since mechanical stresses are known to induce microcracks, which increase secondary remodeling in the area [71, 72].

It is worth noting that this area of high tensile forces in the femur is highly constrained in the sauropodomorph dinosaur phylogeny, since it is observed in both basal (e.g., *Riojasaurus*) and derived (e.g., *Patagosaurus*) forms. The differences observed in terms of areas affected (i.e., a single area in *Riojasaurus*, and two distinct areas in *Lessemsaurus*) are possibly related with variations in the locomotory habits of these taxa. If this hypothesis is correct, the presence of a single “radial patch” of modified primary bone in non sauropodiform sauropodomorphs (i.e. *Riojasaurus* and *Coloradisaurus*) could be related to phylogenetic variations in the locomotory habits of sauropodomorph dinosaurs. Following Petermann and Sander [69], the presence of both high density of Sharpey’s fibers and modified vascular pattern in the inner, mid and outer cortex indicates that the pattern of muscle attachment (and the differential tensile forces) were maintained from earlier ontogenetic stages.

Despite the relative abundance of histological studies of sauropodomorph dinosaurs that included femoral thin sections (e.g. [8, 15, 16, 55–59, 73–75]), it is intriguing that the variation in the distribution of Sharpey’s fibers and the concurrent change in the vascularization pattern is not mentioned. Perhaps this is because slightly different parts of the shaft were studied, cores were used instead of complete transverse sections, and/or complete secondary remodeling of the cortex had occurred. To our knowledge, radial patches of secondary remodeling in the dinosaur long bones have only been reported by Reid [76] in an *Iguanodon* femur.

## Concluding remarks

The bone histology of the ten sauropodomorph dinosaurs studied here, as well as an assessment of the published literature on the histology of sauropodomorph dinosaurs representing more than 25 taxa, provides novel insight pertaining to the paleobiology of this clade. Our results reveal significant variation in the growth dynamics of the Sauropodomorpha, and show that uninterrupted rapid rates of growth evolved independently in both basal and derived forms. Moreover, we found that despite its large body size, the basal sauropod *Lessemsaurus* exhibits the plesiomorphic sauropodomorph pattern of cyclical growth dynamics. Along with its more basal growth strategy, *Lessemsaurus* also exhibits other morphological (e.g., absence of pleurocels in the cervical vertebrae) and microanatomical (e.g., absence of cancellous bone in the marrow cavity) plesiomorphic features. This mosaic of features evident in the transition from basal sauropodomorph to the early Sauropoda, reinforces the “transitional” nature of *Lessemsaurus* in the evolution of the lineage. According to Sander et al. [3], gigantism in sauropod dinosaurs was attained through an uninterrupted rapid growth strategy. However, our findings show that only the derived sauropodomorpha (i.e. Eusauropoda) exploited rapid, uninterrupted, growth during early ontogeny to obtain gigantic proportions. Furthermore, our study shows that the sauropodomorpha exploited both cyclical and uninterrupted growth strategies to achieve large body size.
